# The Nuclear Receptor NR1B1/RARα Arrests the Differentiation of Anti‐Tumor Effector Cytotoxic T Cells

**DOI:** 10.1002/advs.202410241

**Published:** 2025-03-11

**Authors:** Patrick Niekamp, Ryun Hee Kim, Adithyan Jayaraman, Nils Klement, Raymond Kostlan, Chang H. Kim

**Affiliations:** ^1^ Department of Pathology University of Michigan School of Medicine Ann Arbor MI 48109 USA; ^2^ Mary H. Weiser Food Allergy Center University of Michigan School of Medicine Ann Arbor MI 48109 USA; ^3^ University of Bielefeld Faculty of Physics 33615 Bielefeld Germany; ^4^ Immunology Graduate Program University of Michigan Ann Arbor MI 48109 USA; ^5^ Rogel Cancer Center University of Michigan School of Medicine Ann Arbor MI 48109 USA

**Keywords:** BATF, CD8 T cells, histone deacetylase, RARα, retinoic acid, trafficking receptor switch, tumors

## Abstract

NR1B1/RARα expression is dynamically regulated in cytotoxic lymphocytes (CTLs) in tumors, but the importance of its expression in anti‐tumor CTLs remains unknown. RARα gene expression is upregulated in CTLs in tumor microenvironments (TME), but its protein expression is downregulated by retinoic acid. The role of RARα expression in regulating anti‐tumor effector CTL (Teff) differentiation is reported. Mice that over‐express RARα in T cells are defective in early Teff differentiation and fail to populate tumors. In contrast, RARα‐deficient CTLs are hyper‐active in making tumor‐populating Teff cells, suggesting that RARα represses Teff differentiation. Moreover, RARα negatively controls the trafficking receptor switch from the lymphoid to an effector type. Generation of chimeric antigen receptor (CAR) T cells with reduced RARα expression produces highly effective CAR T cells with enhanced anti‐tumor cytotoxicity. Mechanistically, upregulated RARα expression decreases the nuclear histone acetylase (HAT) activity, required for TCF1 to BATF transcription factor and trafficking switches during Teff differentiation. Additionally, RARα and BATF closely associate with each other on Teff‐associated genes on the chromatin for possible cross‐regulation. In sum, T cell‐expressed RARα is identified as a novel negative regulator and potential target of intervention in promoting anti‐cancer T cell immunity.

## Graphical abstract

RARα is highly expressed by differentiated effector and exhausted T cells and exerts a negative effect on effector T cell differentiation and the associated trafficking receptor switch. Upregulated RARα levels decrease the nuclear histone acetylase (HAT) activity, and this suppresses the expression of key Teff transcription factor BATF and trafficking receptors potentially important for migration into tumors.

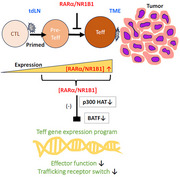

## 1 Introduction

Cytotoxic T lymphocytes (CTLs) become functional effector cytotoxic T (Teff) cells following their activation and differentiation from naïve or memory CTLs. This process is regulated by numerous factors including antigen stimulation, types of pathogens, co‐stimulatory ligands, cytokines, nutrients, and endocrine hormones.^[^ ^1^ ^]^ In many chronic pathological conditions such as cancer and chronic infections, however, CTLs assume non‐functional or exhausted phenotypes due to repeated T cell receptor (TCR) stimulation and deficiencies in positive but increased levels of negative stimulation signals.^[^ ^2^ ^]^ In this regard, certain macronutrients and micronutrients are considered important regulators of Teff differentiation.^[^ ^3^ ^]^

CTLs express nuclear receptors (NRs), such as LXRβ/NR1H2, NR4A1, NR4A2, NR4A3, glucocorticoid receptor (GR/NR3C1), and androgen receptor (AR/NR3C4), and these NRs play potent negative roles in effector CTL differentiation in chronic antigen stimulation conditions that induce CTL dysfunction.^[^ ^4^ ^]^ Another prominent NR expressed by CTLs but not well understood in its function is the retinoic acid receptor‐α (RARα/NR1B1). NRs regulate cells largely through their transactivation^[^ ^5^ ^]^ and transrepression^[^ ^6^ ^]^ functions. The transactivation function involves NR binding to specific DNA motifs, such as retinoic acid response elements (RAREs) for RARα as an example. Among NRs, RARα is triggered by retinoic acid (At‐RA, hereafter called RA), which is produced from retinol (commonly called vitamin A). In the absence of RA, RARα is associated with NCoR/SMRT/histone deacetylase (HDAC) co‐repressor complexes, which condense the chromatin and prevent transcription.^[^ ^7^ ^]^ Upon ligand binding, RARα releases the co‐repressor complexes but recruits coactivator complexes which include p300/CBP histone acetyltransferase (HAT) for histone modifications.^[^ ^8^ ^]^ This is followed by recruitment of ATP‐dependent chromatin remodelers (SWI/SNF) to reposition nucleosomes conducive for gene expression. Additionally, the transrepression function of RARα is mediated through its negative effect on other transcriptional factors, such the AP‐1 proteins.^[^ ^7^ ^]^ In addition, non‐genomic and ligand‐independent rapid activation of T cell signaling by non‐nuclear RARα in CD4 T cells has been reported.^[^ ^9^ ^]^

Despite the progress, the role of RARα in regulating effector CTL (Teff) differentiation remains largely unclear. CTLs play a central role in fighting viral infections and cancers.^[^ ^2a^ ^]^ In this regard, a positive impact of RA on T cell responses has been described.^[^ ^10^ ^]^ In addition, RA induces the expression of the gut‐homing receptors CCR9 and α4β7.^[^ ^11^ ^]^ Vitamin A metabolites and RA signaling are known to increase the expression of granzyme B and perforin to increase CTL activity in acute conditions.^[^ ^10^, ^12^ ^]^ RARα was initially implicated in supporting an acute CTL response to infection,^[^ ^12b^ ^]^ but the intrinsic function of RARα in chronic conditions, such as tumor microenvironments (TME), remains unknown.

We investigated the expression of RARα and its function in regulating Teff CTL differentiation in TME. Our data indicate that upregulated RARα, unexpectedly, has a potent negative effect on Teff differentiation in TME. In contrast, decreased RARα expression promoted anti‐tumor Teff cells in tumors. We found that RARα restrains the nuclear HAT activity, required for Teff differentiation processes, such as the TCF1 to BATF transcriptional factor and trafficking receptor switches in CTLs. We also demonstrated that the strategy to boost Teff CTL activity by down‐regulating RARα expression can be applied to boost the performance of chimeric antigen receptor (CAR) T cell therapies. Our results establish RARα as a potent negative regulator of Teff differentiation in tumors and a functionally important target to invigorate anti‐tumor CTL activity.

## 2 Results

### 2.1 The Expression of RARα in CTLs is Dynamically Regulated by Differentiation Status and Ligand Concentration

**Figure 1:**
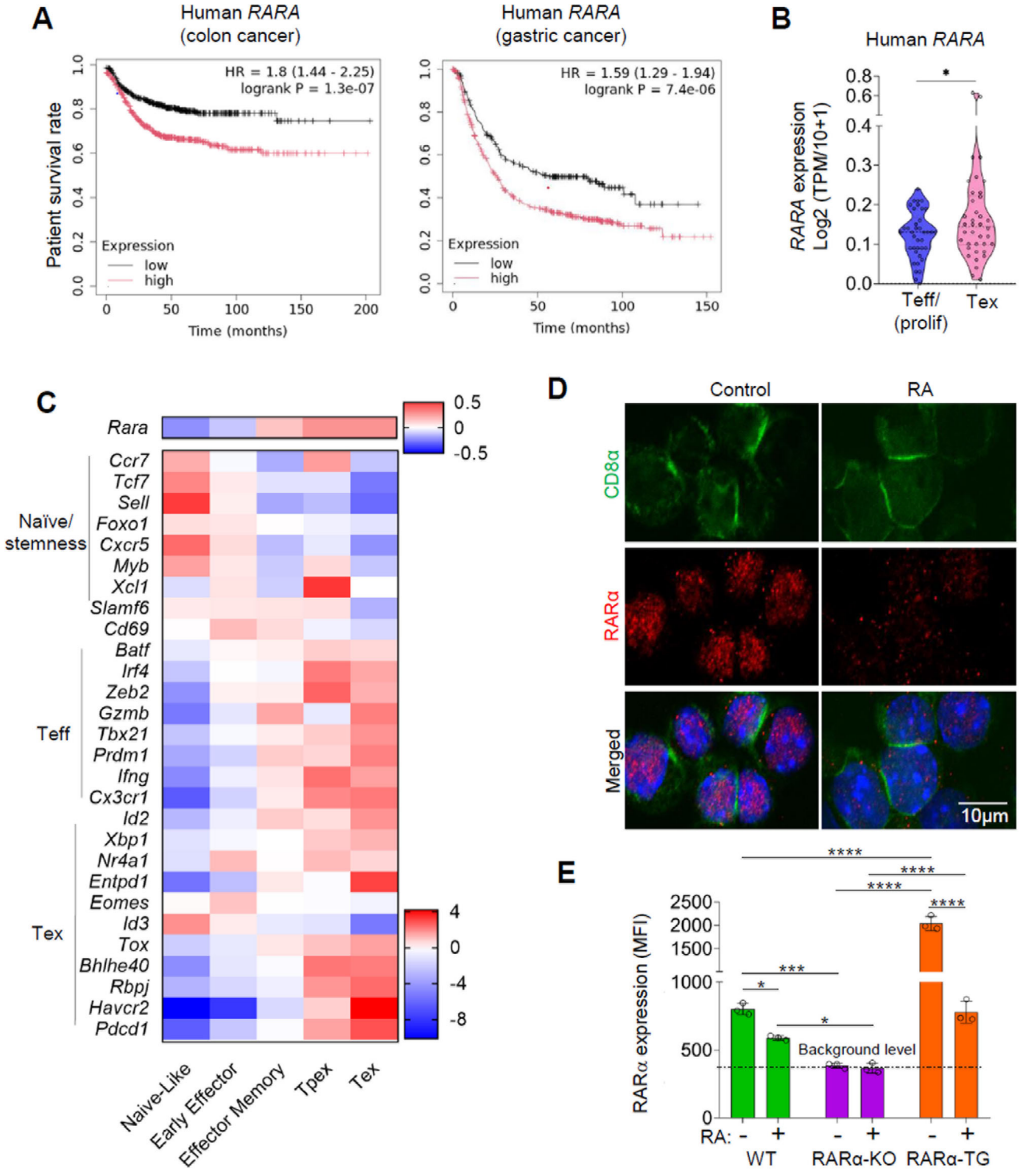
Dynamic regulation of RARα gene and protein expression levels in CD8 T cells during differentiation and by RA. (A) Patient survival data for colon cancer and gastric cancer. The data were retrieved from the KM plotter using the gene expression data and relapse‐free and overall survival data from GEO, EGA, and TCGA. (B) *RARA* expression in tumor‐infiltrating CTL subsets in 16 human cancer types. A violin plot showing the log2‐fold expression of human *RARA* in *CD8^+^* proliferating (Teff/prolif) non‐Tex and *CD8* ^+^ exhausted (Tex) cells from 38 publicly available datasets derived from the Tumor Immune Single‐cell Hub 2 (TISCH2).^[^ ^15^ ^]^ (C) A heatmap showing the log2‐fold change expression of *Rara* and genes associated CTL differentiation in pooled scRNA‐seq data for six murine melanoma and colon adenocarcinoma samples.^[^ ^14^ ^]^ (D,E) RARα protein expression in CD8 T cells that were cultured with or without RA. Naïve CD8 T cells were isolated from the spleen of WT, RARα‐KO, and RARα‐TG mice and cultured for 12 h under T cell activation conditions with anti‐CD3, anti‐CD28, and IL‐2 in a RA‐depleted medium with or without RA. Statistical significance was determined using two‐way ANOVA (E) with Tukey's multiple comparison test. ^*^ *p* ≤ 0.05; ^**^ *p* ≤ 0.01; ^***^ *p* ≤ 0.001; ^****^ *p* ≤ 0.0001.

While RARα agonists and antagonists have been studied for decades for their effects on cancer, the expression and role of RARα itself in regulating cancer, particularly affecting anti‐tumor T cell responses, have been unclear. Our analysis of publicly available data,^[^ ^13^ ^]^ revealed that the expression of *RARA*, the gene coding RARα, is inversely associated with cancer patient survival (**Figure** **1**A). Recent single‐cell RNA‐seq studies revealed that CTLs in tumors are heterogenous in proliferation, differentiation, and effector function and composed of various subsets, including early proliferating cells, Teff, precursor exhausted cells (Tpex), and exhausted cells (Tex).^[^ ^14^ ^]^ Using publicly available data sets,^[^ ^14^, ^15^ ^]^ we found that *RARA* expression is higher in human Tex compared to non‐Tex proliferating CTLs in tumors (Figure 1B). Similarly, *Rara* expression was low in naive‐like and early effector CTLs but up‐regulated in more differentiated effector memory, Tpex, and Tex CTL subsets in mouse MC38 tumors (Figure 1C).^[^ ^14^ ^]^ Thus, RARα gene expression changes in CTLs according to their differentiation or exhaustion stages.

RA is produced by tumors, presumably at various levels depending on tissues and cancer types.^[^ ^12^, ^16^ ^]^ RA rapidly induces RARα modifications (phosphorylation, SUMOylation, and/or ubiquitination) in many cell types including T cells.^[^ ^17^ ^]^ Particularly, RARα is tagged for degradation by murine double minute‐2 as an E3 ubiquitin ligase for degradation by the ubiquitin‐proteasome pathway.^[^ ^18^ ^]^ Thus, tissue‐produced RA would affect the protein level of RARα in cells. Because this has not been examined in immune cells, particularly CTLs, we examined the impact of RA on the level of RARα expression in CTLs during T cell activation (Figure 1D). We utilized RARα‐KO (Lck‐Cre×Rara fl/fl) as a negative control and RARα‐TG (hCD2‐Rara) CTLs for improved detection of RARα in addition to control WT CTLs.^[^ ^9a^ ^]^ We found that RA effectively decreased the expression of RARα at protein level in WT and TG CTLs in culture for 12 h (Figure 1D,E; Figure S1, Supporting Information). To gain more insight into the potential degradation process, we utilized inhibitors of 26S proteasome (R‐MG132 and S‐MG132), lysosomal (chloroquine), and calpain (calpain inhibitor II) pathways in the CTL culture. We found none of these inhibitors were able to suppress the RA‐induced degradation of RARα (Figure S1, Supporting Information). Thus, a pathway other than the three major pathways could be involved.

Overall, these data indicate that RARα expression is dynamically regulated in CTLs depending on intrinsic (differentiation) and extrinsic (ligand levels) factors. The information identified a need to study the impact of up‐ or down‐regulated expression of RARα on CTL differentiation in tumors. To be used as animal models for the study, we tested and verified that the RARα‐KO and RARα‐TG mice described in Figure 1E produced CTLs with decreased or increased expression of RARα, respectively, in B16 tumors (Figure S2, Supporting Information).

### 2.2 Reciprocal Effects of RARα Overexpression Versus Deletion in T Cells on Tumor Growth

**Figure 2:**
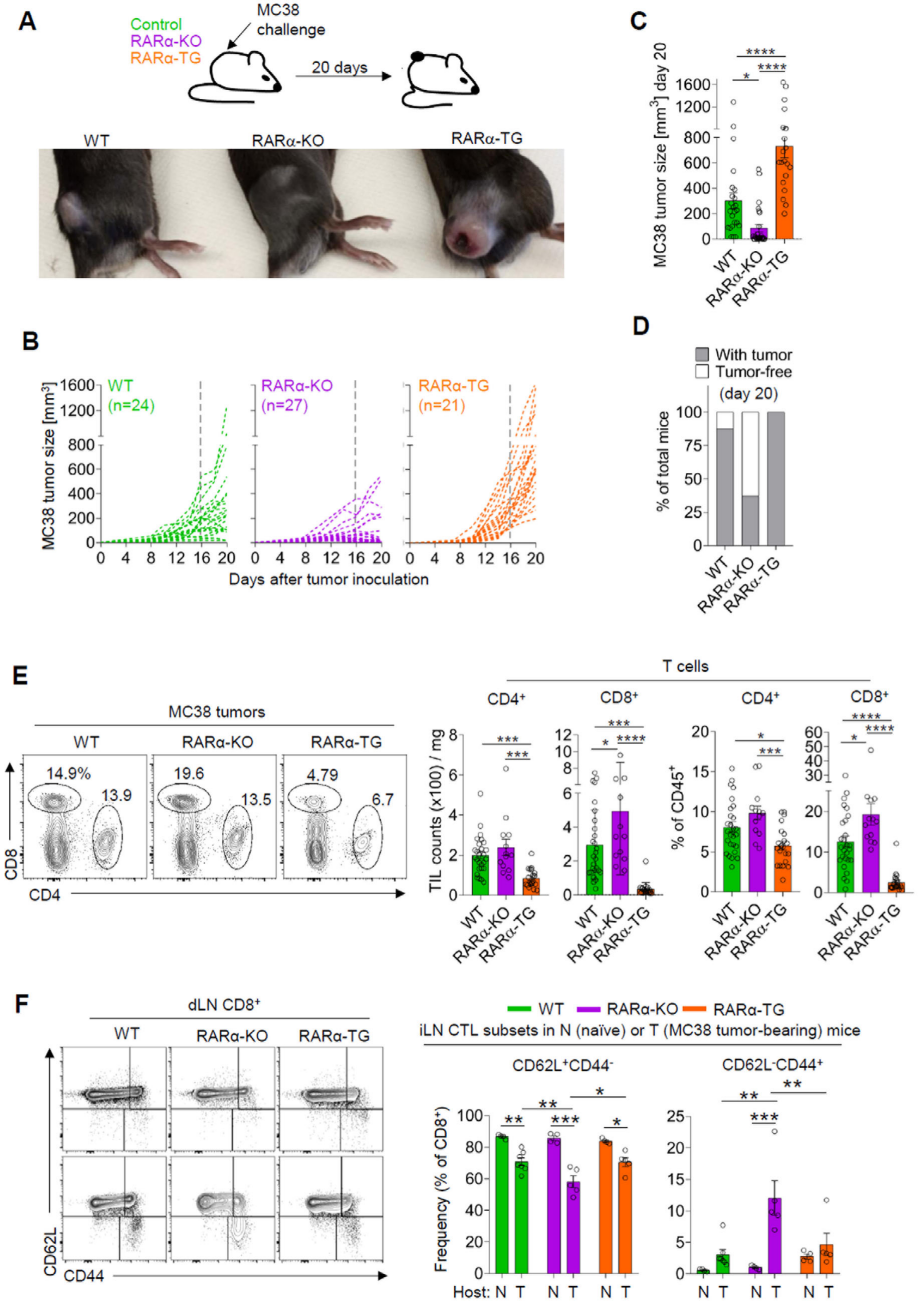
The impact of up‐ or down‐regulated RARα on tumor growth, T cell infiltration in tumors, and Teff CTLs in tumor‐draining lymph nodes. (A) MC38 tumor growth in WT, RARα‐KO, and RARα‐TG mice. Representative images are shown. (B) Growth curves of MC38 tumors. (C) Tumor sizes on day 20 post‐tumor inoculation. (D) Frequencies of tumor‐bearing and tumor‐free mice on day 20. (E) Flow cytometry data showing the frequency and absolute counts of CD4 and CD8 T cells among tumor‐infiltrating CD45^+^ cells in WT, RARα‐KO, and RARα‐TG mice. (F) Numbers and frequencies of CD62L^+^CD44^−^ and CD62L^−^CD44^+^ T cells in the draining lymph nodes of control, RARα‐KO, and RARα‐TG mice. Data are means ± SEM. n ≥ 13 mice. Statistical significance was determined using one‐way ANOVA (C,E) or two‐way ANOVA (F) with Tukey's multiple comparison test. ^*^ *p* ≤ 0.05; ^**^ *p* ≤ 0.01; ^***^ *p* ≤ 0.001; ^****^ *p* ≤ 0.0001.

To determine the impact of the varied expression of RARα in T cells on tumor growth, we subcutaneously implanted MC38 tumor cells into control, RARα‐KO, and RARα‐TG mice (**Figure** **2**). Interestingly, the growth of MC38 tumors in RARα‐KO mice was considerably slower than those in WT mice (Figure 2A–C). Moreover, ≈60% of RARα‐KO mice became tumor‐free by day 20 (Figure 2D). In contrast, MC38 tumor growth was greatly accelerated in RARα‐TG mice, often developing ulcers on tumors. We also found significant differences between RARα‐KO and RARα‐TG mice in the growth of more aggressive B16 tumors. RARα‐KO mice were slower in reaching a significant size (700 mm^3^) of tumors compared to WT and RARα‐TG mice on day 16 (Figure S3A,B, Supporting Information). Overall, the negative effect of RARα on tumor growth was detected in the two types of tumors, but it was greater on MC38 than B16 tumors. To verify that the process is driven by T cells, but not other types of cells, we transferred spleen T cells, isolated from WT, RARα‐KO, or RARα‐TG mice into MC38‐bearing Rag1‐KO mice (Figure S3C, Supporting Information). While less pronounced in the Rag1‐KO background, a similar trend of decreased and increased tumor growth was observed in the mice transferred with RARα‐KO T cells and RARα‐TG T cells. Thus, RARα deficiency in T cells suppressed tumor growth, whereas over‐expressed RARα increased the growth. The efficient rejection of MC38 tumors in RARα‐KO mice was interesting, and we further determined if the RARα‐KO mice would develop a memory T cell response for efficient rejection of tumors upon re‐challenge. We implanted the same tumor cells into naïve versus previously tumor‐rejected RARα‐KO mice (Figure S3D,E, Supporting Information). Tumors grew in tumor‐naïve mice but did not grow (8/10 mice) or quickly regressed (2/10 mice) in previously tumor‐rejected RARα‐KO mice. Thus, RARα‐KO mice develop an effective memory response to tumors. We observed similar patterns of tumor growth in both male and female mice (not shown) and most of the subsequent studies were performed on male mice for consistency.

### 2.3 Upregulated T Cell‐Expressed RARα Arrests Teff Differentiation in TME

Next, we examined tumor infiltration by T cells in WT, RARα‐KO, and RARα‐TG mice. We observed distinct changes in numbers and frequencies of CTL populations in MC38 or B16 tumors of RARα‐KO versus RARα‐TG mice (Figure 2E; Figure S3F, Supporting Information). The numbers of CD4 and CD8 T cells were higher in RARα‐KO, but lower in RARα‐TG, mice compared to WT mice. These differences were consistently observed at two different times (the early 15–18‐day and the late 20–25‐day periods post‐tumor injection, not shown). In contrast, the CTL frequency in the tumor‐draining lymph nodes (dLN) of RARα‐KO mice was notably decreased compared to that of WT and TG mice (Figure S4A,B, Supporting Information). The frequency of naïve‐like CD62L^+^CD44^−^ CTLs was decreased, whereas that of more differentiated CD62L^−^CD44^+^ effector‐like CTLs was increased in the dLN of RARα‐KO mice (Figure 2F). A similar difference was observed for CD4^+^ T cells (Figure S4C,D, Supporting Information). In addition, we examined the frequencies of Tex and Tpex in dLNs and tumors and found that most of CTLs were not Tpex or Tex in this tumor model, and there was no significant difference in their numbers among the mouse lines (Figure S5A,B, Supporting Information). We examined also the frequencies of FoxP3^+^ Tregs in the tumors of these mice because Tregs are implicated in the regulation of anti‐tumor immune responses.^[^ ^4a^ ^]^ Despite small differences, such as an increase in RARα‐KO in MC38 tumors and a decrease in RARα‐TG in B16 tumors, Treg frequencies among CD4 T cells in the dLN and tumors of RARα‐KO and RARα‐TG were largely comparable (Figure S6A,B, Supporting Information).

**Figure 3:**
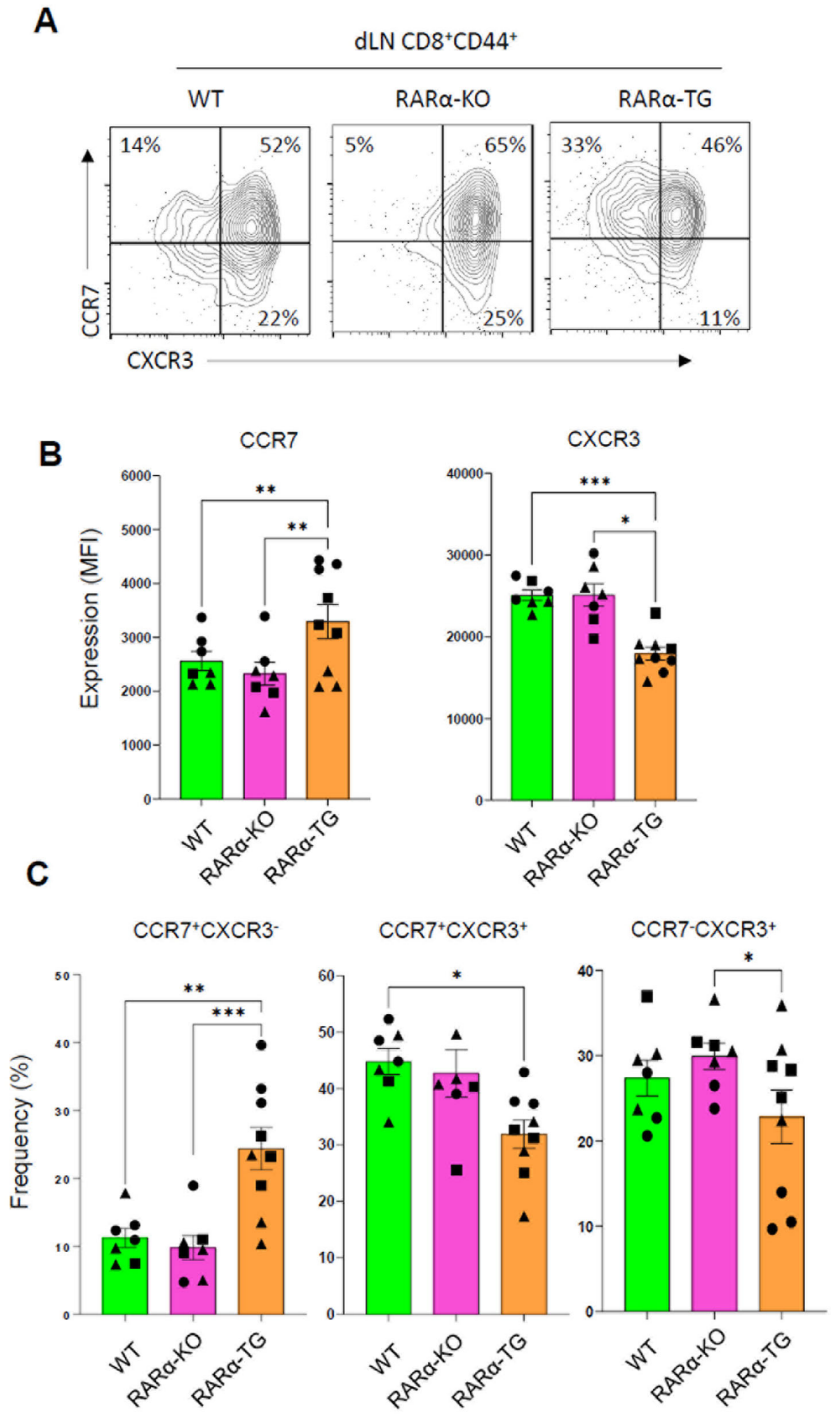
The negative impact of RARα expression on the Teff trafficking receptor switch in tumor‐draining lymph nodes. (A,B) Expression of CCR7 and CXCR3 by CD44^+^ CTLs in the dLNs of MC38 tumor‐bearing control, RARα‐KO, and RARα‐TG mice. (C) Frequencies of CCR7^+^CXCR3^−^ and CCR7^−^CXCR3^+^ CTLs in the dLNs of MC38 tumor‐bearing control, RARα‐KO, and RARα‐TG mice on 15–25 days post tumor implantation. Flow cytometry was performed. Statistical significance was determined using one‐way ANOVA with Tukey's multiple comparisons test. ^*^ *p* ≤ 0.05; ^**^ *p* ≤ 0.01; ^***^ *p* ≤ 0.001; ^****^ *p* ≤ 0.0001.

When we examined the CTL subsets in the repeated challenged RARα‐KO mice (Figure S3D, Supporting Information), these mice did not accumulate the CD62L^−^CD44^+^ effector‐like CTLs in dLNs (Figure S7, Supporting Information), suggesting that the effector T cells probably moved out to effector sites following their generation. This migration of CTLs requires a tracking receptor switch from lymphoid tissue‐ (CD62L and CCR7) to effector site‐homing receptors (e.g., CXCR3, CCR8, CCR5, CCR2, and CCR1) upon antigen priming.^[^ ^19^ ^]^ In this regard, we further examined the expression of CCR7 and CXCR3 (a major Teff‐associated chemokine receptor) by CTLs in the dLNs of tumor‐bearing control, RARα‐KO, and RARα‐TG mice (**Figure** **3**A–C). We found that RARα‐TG mice had decreased frequencies of CXCR3^+^ CTLs but increased frequencies of unswitched CCR7^+^CXCR3^−^ CTLs in dLNs, suggesting a defect in the trafficking receptor switch. Particularly, RARα‐TG mice had significant differences from RARα‐KO mice in the frequencies of CCR7^+^CXCR3^−^ and CCR7^−^CXCR3^+^ CTLs in dLNs. Thus, these data indicate that the trafficking receptor switch during Teff differentiation is suppressed by upregulated RARα, which is in line with the relatively low infiltration of CTLs in tumors of RARα‐TG mice.

**Figure 4:**
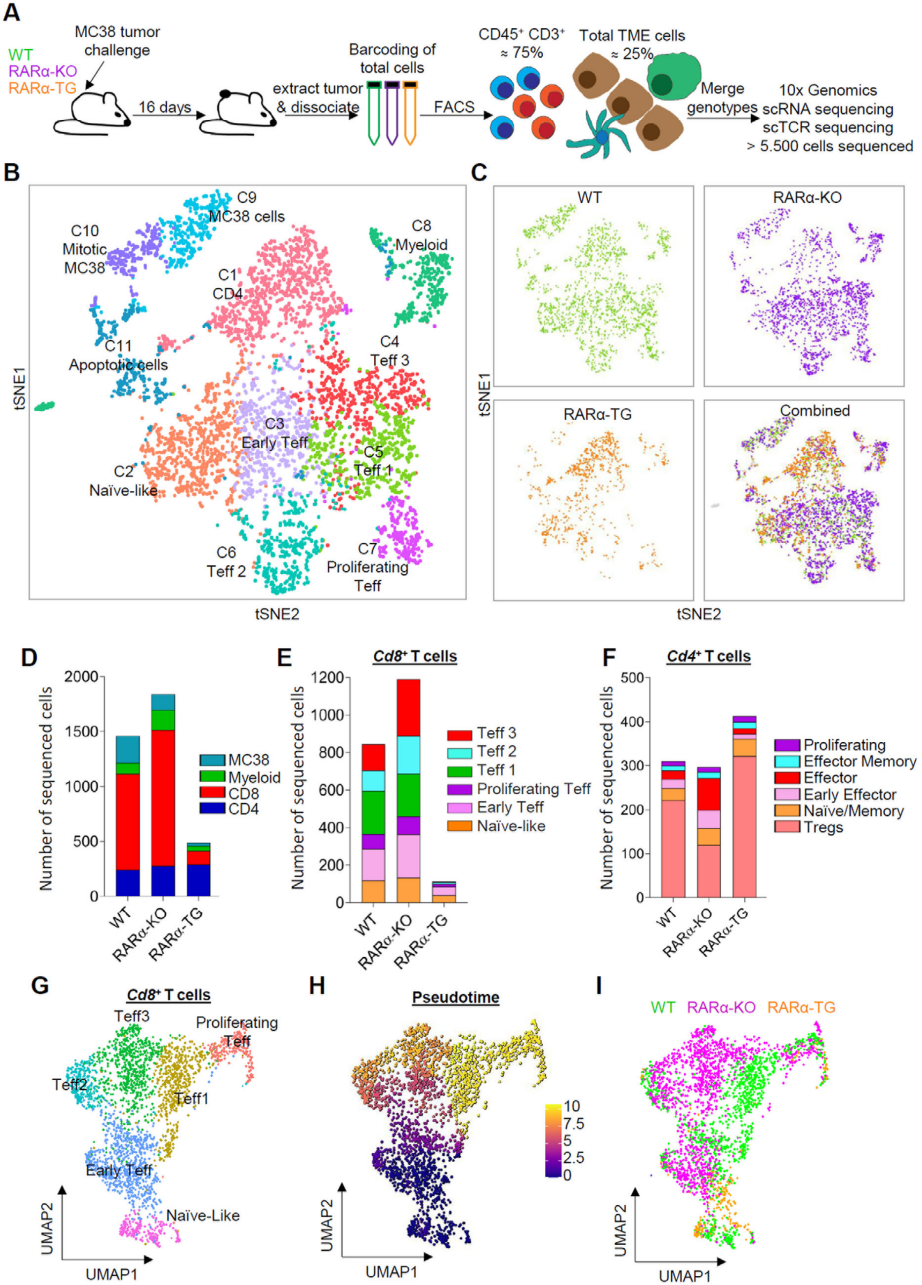
T cell‐expressed RARα decreases Teff CTLs in tumors. (A) The experimental design for the multiplexed scRNA‐seq with TCR repertoire analyses for remixed T cell‐enriched tumor cell samples after cell sorting. (B) A tSNE plot of sequenced cells showing different cell clusters. (C) tSNE plots showing distribution of sequenced cells for WT, RARα‐KO, RARα‐TG, and combined samples. (D) Predicted overall cell compositions for MC38 tumors of WT, RARα‐KO, and RARα‐TG mice based on tSNE cluster information. (E) Predicted CTL compositions in the MC38 tumors of WT, RARα‐KO, and RARα‐TG mice based on tSNE cluster information. (F) Predicted CD4^+^ T cell compositions in the MC38 tumors of WT, RARα‐KO, and RARα‐TG mice based on tSNE cluster information. (G) A UMAP plot showing the 2D distribution of the re‐clustered CTL subsets. (H) A UMAP plot showing differentiation trajectory inferred by Monocle3. (I) A UMAP plot showing differential distribution of TIL CTLs from MC38 tumors of WT, RARα‐KO, and RARα‐TG mice.

To gain unbiased information on not only T cells but also other tumor‐infiltrating immune cells such as macrophages and dendritic cells (DCs), we performed single‐cell RNA sequencing (scRNA‐seq) for enriched CD45^+^ CD3^+^ tumor‐infiltrating cells combined with non‐T cells in tumors at a 3:1 ratio (**Figure** **4**A). Unsupervised t‐Distributed Stochastic Neighbor Embedding (tSNE) clustering analysis identified eleven cell clusters (C1–C11) (Figure 4B; Figure S8A,B, Supporting Information). C1 was identified as CD4 T cells. The C2‐C7 clusters were largely composed of conventional CTLs with some CD4 T cells (Figure S8B, Supporting Information). Higher expression of *Rara* in RARα‐TG T cells compared to WT CTLs was detected (Figure S8C, Supporting Information). *Rara* transcripts were still detected in RARα‐KO T cells, because the mouse line still expresses a *Rara* transcript that lacks exon 8 in T cells.^[^ ^20^ ^]^ The most apparent difference was the differential frequencies of total CTLs in the tumors of RARα‐TG and RARα‐KO mice, which, respectively, had decreased and increased levels of CTLs compared to those of WT mice (Figure 4C,D). Among CTLs, early‐stage activated CTLs with a naïve cell phenotype (C2; high expression of *Ccr7* and *S1pr1*), early Teff cells (C3–C7) with both naïve and effector phenotype, and fully differentiated Teff cells were detected (Figure S8B, Supporting Information). The expression of common CTL exhaustion markers, such as *Tox, Pdcd1, Bhlhe40, Rbpj*, and *Havcr2*, was detected on these Teffs, but their expression was too moderate to call them Tpex or Tex (Figure S8B, Supporting Information). This is in line with the low frequencies of Tpex and Tex in dLNs and tumors (Figure S5, Supporting Information). The tumors in the three different mouse groups (i.e., WT, RARα‐KO, and RARα‐TG) had distinctive compositions of tumor‐infiltrating T cell clusters. Early Teff cells and Teff 1–3 subsets were increased in the tumors of RARα‐KO mice (Figure 4E). While all CTLs were decreased in MC38 tumors of RARα‐TG mice, the most severely decreased CTL subsets were Teff 1–3. Compared to CTLs, CD4 T cells were mildly affected, with increased effector but decreased Tregs in RARα‐KO tumors compared to RARα‐TG tumors (Figure 4D,F). A pseudotime trajectory analysis of scRNA‐seq data confirmed the differentiation status of the CTL clusters based on their gene expression and the impact of RARα expression on the composition of the CTL clusters (Figure 4G–I). WT and RARα‐KO CTLs were found at all of the pseudotime stages, but the early effector and Teff 3 subsets were more enriched in RARα‐KO compared to WT CTLs, suggesting significant differences in gene expression. In contrast, most of the few RARα‐TG CTLs were found mainly at the early and proliferating, but not Teff, pseudotime stages, implying profound defects in CTL differentiation (Figure 4G–I). Overall, the RARα level in T cells apparently controls tumor T cell composition, with high and low levels of RARα, respectively, causing a defect and a boost in Teff enrichment in tumors.

### 2.4 RARα Restrains the Clonotypic Expansion of CTLs in Tumors

**Figure 5:**
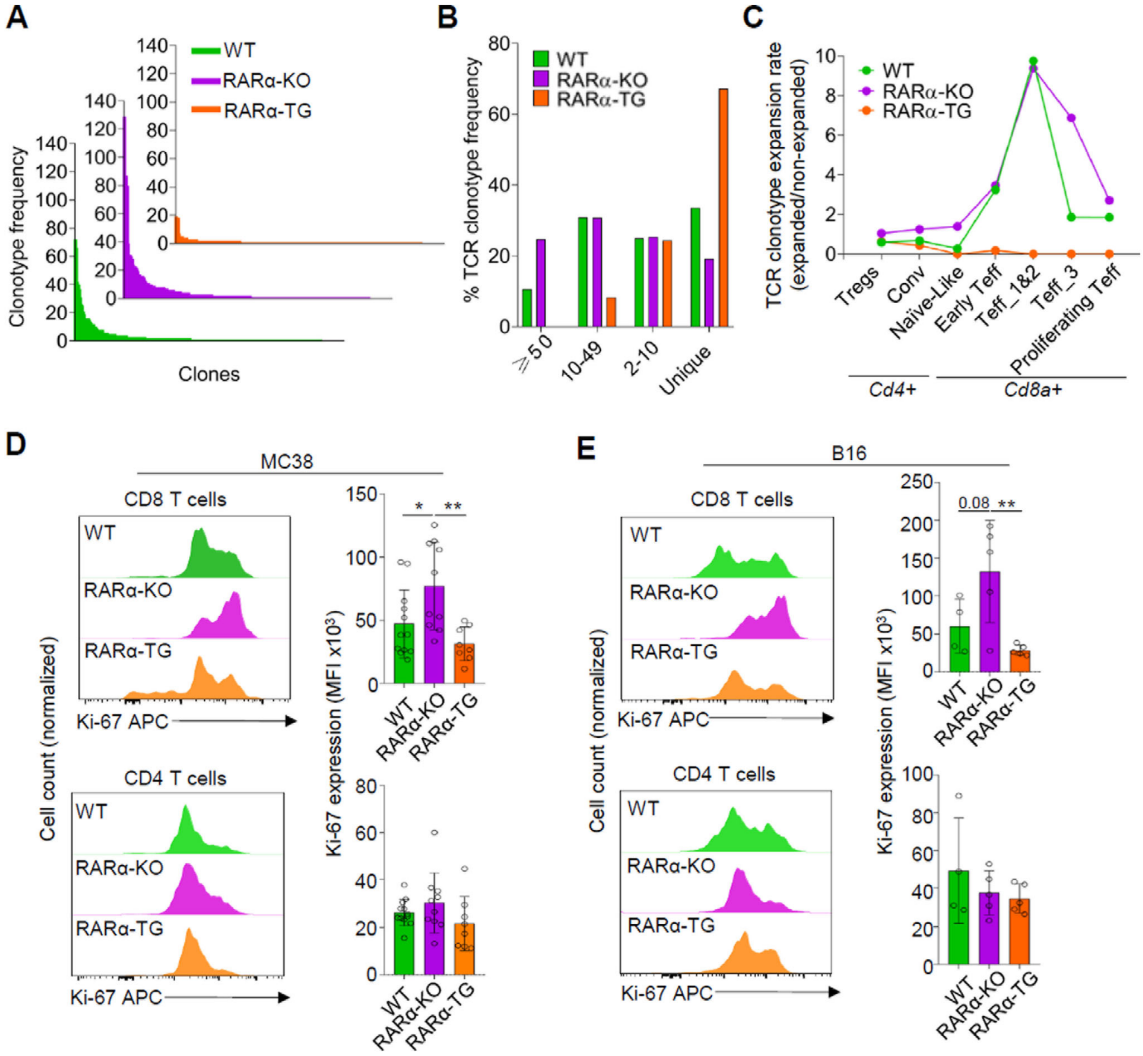
T cell‐expressed RARα suppresses the CTL clonal expansion in tumors. (A) T cell clonotype frequency in MC38 tumors of WT, RARα‐KO, and RARα‐TG mice. (B) Composition of TCR clones based on clonotype frequency (≥50, 10–49, 2–10 or 1) for T cells in MC38 tumors. (C) TCR clonotype expansion by CD4 and CD8 T cell clusters in MC38 tumors. The ratios of expanded (≥2) over non‐expanded (= 1) TCR clonotypes are shown for each T cell cluster in Figure 2B. Ki‐67 expression by CD4 and CD8 T cells in MC38 (D) and B16 (E) tumors of WT, RARα‐KO, and RARα‐TG mice. Data are means ± SEM. n ≥ 7 mice per group (D) and n ≥ 4 mice per group (E). Statistical significance was determined using one‐way ANOVA with Tukey's multiple comparison test (D,E). ^*^ *p* ≤ 0.01, ^**^ *p* ≤ 0.01.

Antigen‐dependent proliferation of T cells is required to form effector T cells that eliminate tumors. Because of the differences in T cell infiltration in tumors of WT, RARα‐KO, and RARα‐TG mice, we examined the TCR clonotypes of tumor T cells by examining the sequences of complementarity‐determining region (CDR) 3 of TCR α and β chain at the single cell level. This revealed that T cells in the tumors of RARα‐KO mice had CDR3 sequences with higher‐than‐normal levels of clonal expansion, whereas the T cells in the tumors of RARα‐TG T cells had TCR clones with low frequencies, indicating poor clonal expansion (**Figure** **5**A). ≈40% of WT T cells, 55% of RARα‐KO T cells, and only ≈6% of RARα‐TG T cells had highly expanded TCR clonotypes with a frequency greater than 10 (Figure 5B). In contrast, ≈35% of WT, 17% of RARα‐KO, and 70% of RARα‐TG T cells had unique or non‐expanded TCR clonotypes. We also examined the ratio of the expanded to non‐expanded T cells based on the clonotype frequency for different T cell clusters (Figure 5C). This analysis indicates a severe deficiency in TCR clonal expansion of all effector CTL clusters in RARα‐TG mice. Overall, RARα‐TG CTL showed defective clonal expansion, whereas RARα‐KO CTLs had enhanced clonal expansion. In support of this, we found that the CTLs from RARα‐TG tumors had low expression of Ki‐67, a marker of cell proliferation, compared to their counterparts in WT and RARα‐KO tumors (Figure 5D,E). This was observed for tumor CTLs in both MC38 and B16 tumors. However, we found smaller or no significant differences in the expression of Ki‐67 for the CD4 T cells in the tumors of the different mouse lines. Taken together, these findings suggest that RARα negatively affects the proliferation of CTLs in the tumor microenvironment.

### 2.5 RARα Suppresses the Teff Gene Program in Tumors

**Figure 6:**
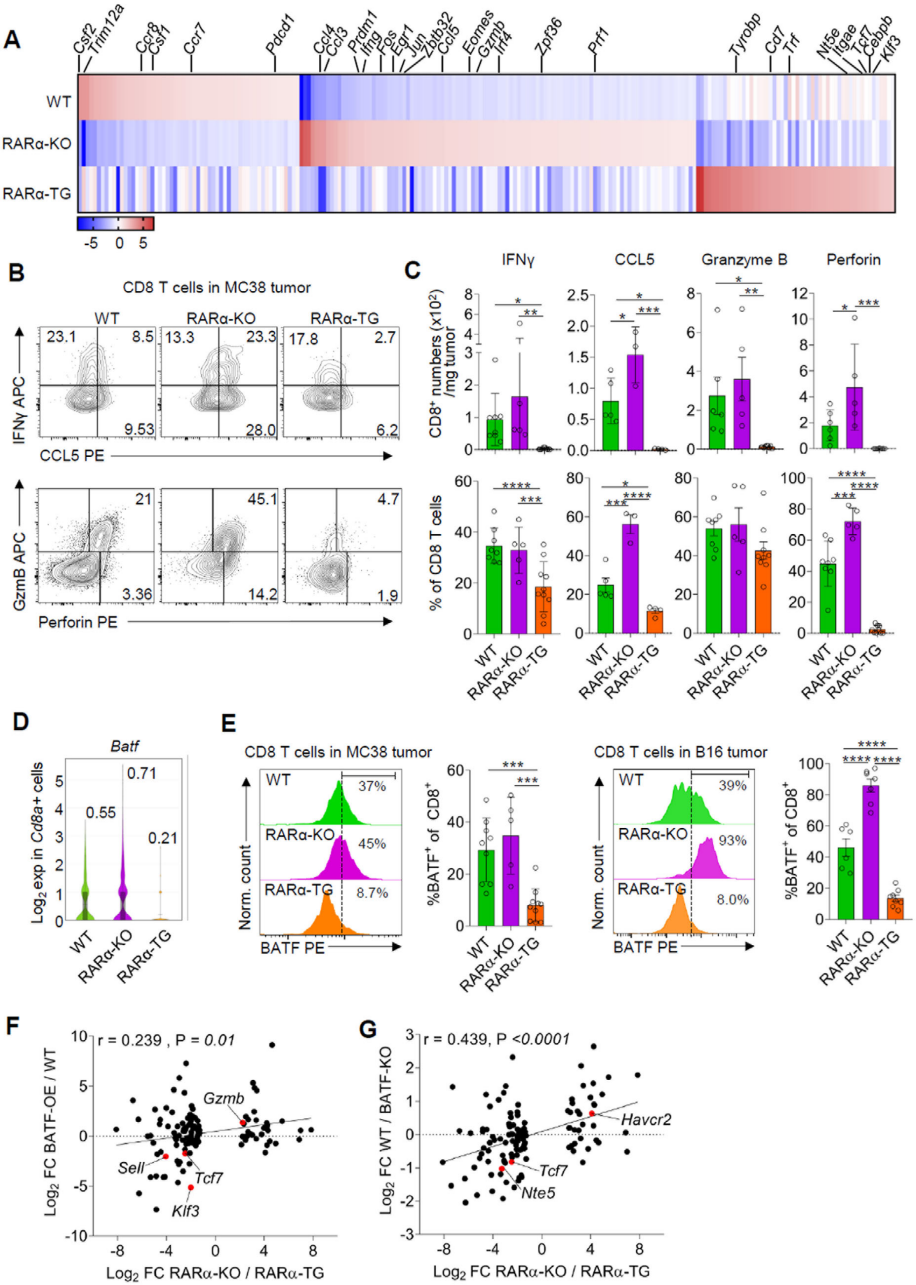
T cell‐expressed RARα suppresses the Teff gene expression program in tumors. (A) A heatmap depicting DEGs from the scRNA‐seq analysis for CTLs in MC38 tumors of WT, RARα‐KO, and RARα‐TG mice. Genes with an average count of ≤0.1 were excluded. Data from 845 WT, 1189 RARα‐KO, and 113 RARα‐TG cells were analyzed. (B) Representative flow data for the expression of IFNγ, CCL5, GzmB, and Perforin by CTLs in MC38 tumors on WT, RARα‐KO, and RARα‐TG mice. (C) Total number (top) and frequency (bottom) of tumor‐infiltrating CTLs expressing IFNγ, CCL5, GzmB, and Perforin as in panel B. Data are means ± SEM. n ≥ 3 mice. (D) BATF expression at mRNA level by CTLs in MC38 tumors based on the scRNA‐seq data. (E) BATF protein expression by CTLs in MC38 and B16 tumors. (F) The correlation between the RARα‐suppressed genes in vivo and the over‐expressed BATF‐regulated genes. The RARα‐suppressed genes in vivo are defined as the genes significantly regulated in RARα‐KO versus RARα‐TG *Cd8a* ^+^ T cells in tumors based on scRNA‐seq data. These relative expression data for the RARα‐suppressed genes were plotted against the relative expression data for BATF‐overexpressing (OE) versus WT tumor‐infiltrating CTLs, which were from publicly available data (GEO GSE154747).^[^ ^26b^ ^]^ (G) The correlation between the RARα‐suppressed genes in vivo and the BATF‐regulated genes in vitro. The BATF‐regulated genes in vitro are defined as the relative expression data for WT versus BATF KO CTLs, which were from publicly available scRNA‐seq data (GEO GSE192390).^[^ ^26a^ ^]^ Pearson correlation coefficients were calculated for two sets of data. Statistical significance was determined using one‐way ANOVA with Tukey's multiple comparison test (C,E) or two‐tailed *t‐*test (F,G). ^*^ *p* ≤ 0.05; ^**^ *p* ≤ 0.01; ^***^ *p* ≤ 0.001; ^****^ *p* ≤ 0.0001.

Differentially regulated genes (DEGs) in the CTLs from WT, RARα‐KO, and RARα‐TG mice were examined to understand the impact of RARα on the transcriptome (**Figure** **6**A). Among the DEGs, genes expressed at higher levels in RARα‐KO CTLs included major effector molecules required for anti‐tumor immunity, such as *Ifng, Gzmb*, and *Prf1* (Figure 6A; Figure S9A, Supporting Information). The differential expression of IFNγ, Perforin, GzmB, and CCL5 by CTLs in both MC38 and B16 tumors was verified at protein level by flow cytometry (Figure 6B,C; Figure S9B, Supporting Information). In contrast to CTLs, the tumor‐infiltrating CD4 T cells from the three mouse groups did not have differences in the cytokine expression profile (not shown).

To systematically analyze cellular communication networks in TME, we applied the CellChat analysis^[^ ^21^ ^]^ to our scRNA seq data obtained for MC38 tumor‐infiltrating T cells in WT, RARα‐KO, and RARα‐TG mice (Figure 4). One of the top predicted interaction networks was “CCL (CC chemokine ligand group)” (Figure S10A, Supporting Information). Particularly, *Ccl3, Ccl4*, and *Ccl5* were up‐regulated in RARα‐KO CTLs (Figure S10B, Supporting Information). The genes for their receptors, *Ccr1* and *Ccr5*, known to promote immune cell infiltration in tumors,^[^ ^22^ ^]^ were expressed by myeloid cells (monocytes and macrophages) and Teff cells, respectively (Figure S10C,D, Supporting Information). In line with this, *Ccl3, Ccl4*, and *Ccl5* were predicted to interact with the *Ccr5* expressed by T cell subsets and with the *Ccr1* expressed by myeloid cells (Figure S10E, Supporting Information).

We found additional trafficking receptor genes differentially expressed by CTL subsets of WT, RARα‐KO, and RARα‐TG mice. Overall, RARα‐KO Teff cells (C4 and C5) expressed the effector site‐homing receptors *Cxcr3, Ccr2*, and *Ccr5* at higher levels but the homeostatic homing receptors *Ccr7, Ccr8, Sell*, and *Itgae* at lower levels (Figure S11, Supporting Information). RARα‐TG Teff cells displayed the opposite expression pattern for these receptor genes. This difference in gene expression is in line with the flow cytometry data for the expression of CCR7 and CXCR3 and further supports the negative effect of RARα on the acquisition of Teff‐associated trafficking receptors by CTLs in TME (Figure 3).

Further analysis with DAVID (the database for annotation, visualization, and integrated discovery) identified the category “sequence‐specific double‐stranded DNA binding” as the most enriched molecular pathway among the RARα‐KO upregulated genes (Figure S12A, Supporting Information). Transcription factors made up a substantial percentage (19.4%) of the genes upregulated in RARα‐KO CTLs, but the bulk of these transcription factors were reciprocally down‐regulated in RARα‐TG CTLs. In line with this, a group of genes that were highly increased by RARα‐KO CTLs were transcription factors, such as *Irf4, Jun, Egr1, Prdm1, Fos*, and *Eomes* (Figure 6A), which are important for CTL differentiation.^[^ ^1^, ^23^ ^]^ The differential expression of IRF4 was verified at protein level in tumor‐infiltrating CTLs of mice bearing MC38 or B16 tumors (Figure S12B, Supporting Information). Conversely, “transcriptional repressor activity” was one of the pathways enriched in RARα‐TG CTLs, and genes encoding transcription factors associated with repressor functions,^[^ ^24^ ^]^ such as *Tcf7, Klf3*, and *Cebpb*, were upregulated in RARα‐TG CTLs (Figure 6A). The differential expression of TCF1 was verified in tumor‐infiltrating CTLs of mice bearing MC38 or B16 tumors (Figure S12C, Supporting Information).

BATF has been described as a pioneer factor for the Teff gene expression program.^[^ ^25^ ^]^ Because BATF is expressed at a high level by RARα‐KO, but at an abnormally low level, by RARα‐TG CTLs (Figure 6D,E), BATF could be a functionally important target of RARα‐regulated pathway. We compared the RARα‐regulated genes from our scRNA‐seq data and BATF‐regulated genes in CTLs from publicly available data (Figure 6F,G).^[^ ^26^ ^]^ We found positive correlations between the RARα‐suppressed genes and the BATF‐regulated (induced) genes, identified in both BATF‐overexpressed CTLs and BATF‐KO CTLs. Thus, the down regulation of BATF expression is a potential mechanism for the negative regulation of Teff differentiation by RARα.

Additionally, the RARα expressed in T cells had a significant impact on the tumor myeloid compartment (Figure S13A–C, Supporting Information). M1 and M2 macrophages in tumors are generally associated with tumor suppression and promotion, respectively.^[^ ^27^ ^]^ M1 macrophages were increased in the tumors of RARα‐KO mice, whereas M2 macrophages were increased in the tumors of RARα‐TG mice (Figure S13D, Supporting Information). Overall, the M1 to M2 ratio in tumors was higher in RARα‐KO mice but lower in RARα‐TG mice, compared to that of WT mice (Figure S13E, Supporting Information). In addition, their gene expression was also different (Figure S13F,G, Supporting Information). The up‐regulated genes in tumor‐infiltrating myeloid cells in RARα‐KO mice included *Rnf128, Cd36, Nos2, Prdx1, Cd274*, and *H2‐Q4*, associated with the immune‐activating phenotype. The up‐regulated genes in tumor‐infiltrating myeloid cells in RARα‐TG mice included *Alx5, Edil3, Acy1*, and *Matk*, which are associated with immune‐suppression.^[^ ^28^ ^]^ The DAVID analysis based on DEGs identified potential pathways that are affected. These include lipid transports and metabolism along with anti‐bacterial responses (Figure S13H,I, Supporting Information), which are also relevant for the myeloid cells in tumors.^[^ ^29^ ^]^ These results indicate that the phenotype of tumor macrophages is shaped by T cell‐expressed RARα.

### 2.6 Decreased RARα Expression Invigorates CAR‐T Cell Performance

**Figure 7:**
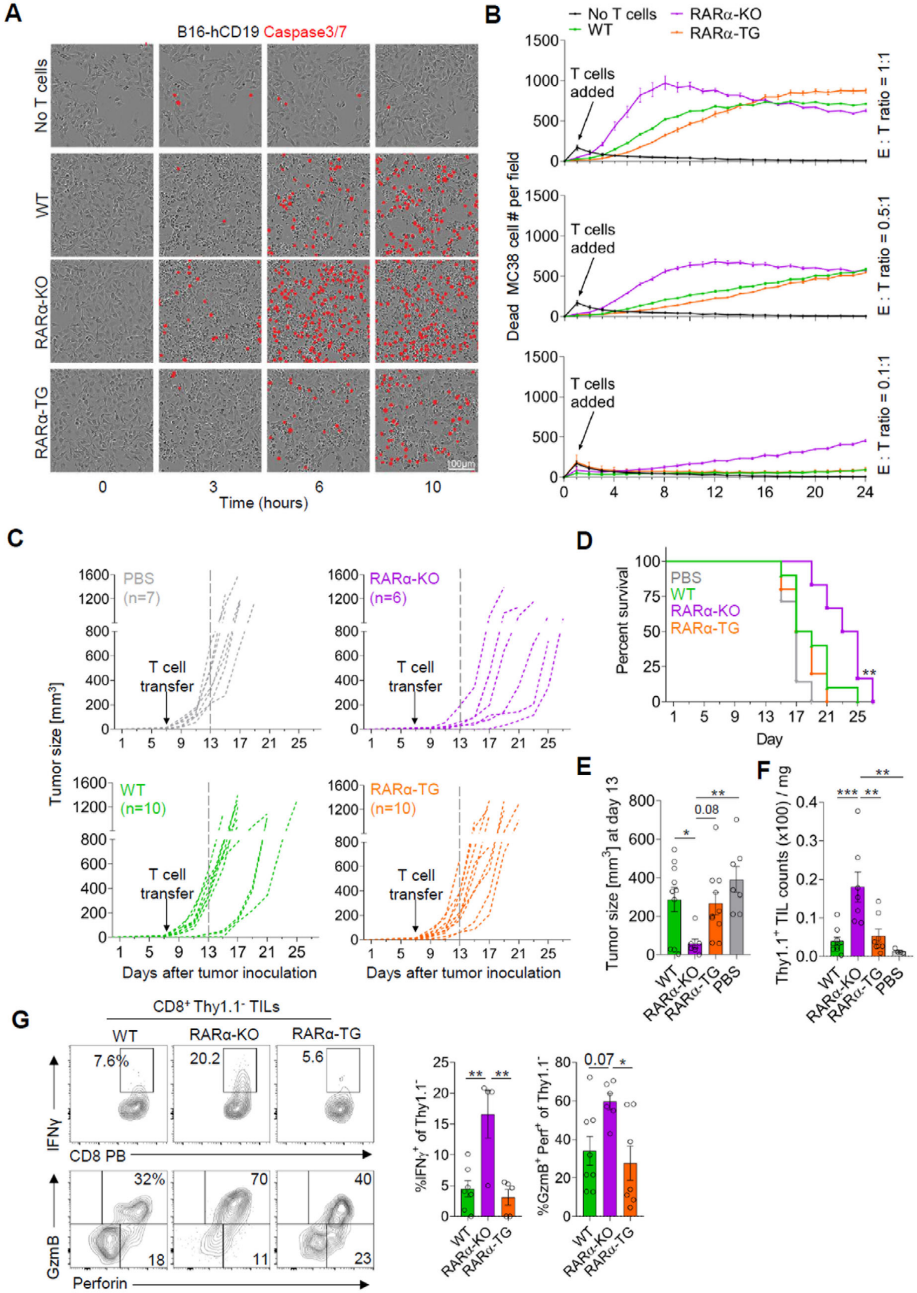
RARα‐deficiency enhances CAR T cell performance. (A) Representative images of cytotoxic activity of WT, RARα‐KO, and RARα‐TG CAR T cells against B16‐hCD19 cells. CAR‐T cells and tumor cells were co‐cultured at 1:1 ratio. Caspase‐positive tumor cells (size ≥ 150 µm^2^) are shown in red. (B) Quantification of cytotoxic activity against B16‐hCD19 over time at different ratios. n ≥ 5 wells over two independent experiments. Error bars denote SEM. (C) Growth of B16‐hCD19 tumor cells in host mice injected with PBS or CAR‐T cells generated from WT, RARα‐KO, or RARα‐TG CTLs. (D) Kaplan–Meier survival curve of B16‐hCD19 tumor‐bearing mice injected with PBS or CAR T cells generated from WT, RARα‐KO, or RARα‐TG CTLs. (E) Tumor size on day 13 post‐tumor injection. (F) Numbers of CD8 Thy1.1^+^ CAR‐T cells in tumors of B16‐hCD19 tumor‐bearing mice. (G) Effector phenotype of bystander non‐CAR‐T tumor‐infiltrating CD8 T cells at experimental endpoints (8–19 days) after the transfer of CAR‐T cells into B16‐hCD19 tumor‐bearing mice. The frequencies of Thy1.1^−^ CD8 T cells expressing IFNγ, GzmB, and/or perforin in B16‐hCD19 tumors of host mice after the transfer of WT, RARα‐KO, or RARα‐TG CAR‐T cells are shown. Data are means ± SEM. n ≥ 4 mice. Statistical significance was determined using log‐rank test (D) or one‐way ANOVA with Tukey's multiple comparison test (E–G); ^*^ *p* ≤ 0.05; ^**^ *p* ≤ 0.01; ^***^ *p* ≤ 0.001.

The increased anti‐tumor T cell activity in RARα deficiency would offer a novel opportunity to boost adoptive T cell therapies. CAR‐T cell therapies are promising but have limitations in durability, efficacy, and immune cell infiltration into solid tumors.^[^ ^30^ ^]^ We reasoned that CAR‐T cells might function better if CAR‐T cell production is combined with RARα deficiency. This approach also allowed us to assess the effect of RARα expression on tumor cells in an antigen‐specific manner. To test this idea, we transduced WT, RARα‐KO, and RARα‐TG CTLs with a retroviral vector expressing a CAR against hCD19 and co‐cultured the cells with hCD19‐expressing B16 or MC38 tumor cells at various effector‐to‐target ratios (**Figure** **7**A,B; Figure S14A–C and Movies S1–S8, Supporting Information). At all the different ratios tested, in vitro cytotoxicity of RARα‐KO CAR‐T cells was higher than that of WT CAR‐T cells. In contrast, RARα‐TG CAR‐T cells were less efficient than WT CAR‐T cells in killing both B16 and MC38 tumor cells expressing hCD19 antigen in vitro.

Next, we determined the anti‐tumor efficacy of WT, RARα‐KO, and RARα‐TG CAR‐T cells in vivo on existing tumors (Figure 7C). After titration experiments, we used 1.5 million CAR‐T cells for B16‐hCD19 tumor‐bearing mice, which is considered a suboptimal number for significant CAR‐T activity for WT CTLs (Figure 7D,E). The B16 tumor model was chosen for this experiment because it is a relatively more aggressive tumor than the MC38 model. RARα‐KO CAR‐T cells were more effective in delaying tumor growth compared to WT or RARα‐TG CAR‐T cells. Thus, RARα deficiency increased anti‐tumor CAR‐T activity. Flow analysis of TILs detected relatively higher numbers of RARα‐KO CAR‐T cells than those of WT and RARα‐TG CAR‐T cells in tumors at the termination of the experiments (Figure 7F). An unexpected but interesting phenomenon was that RARα‐KO CAR‐T cells changed the effector phenotype of host non‐CAR‐T cells (Figure 7G). The host non‐CAR tumor‐infiltrating CTLs in the mice treated with RARα‐KO CAR‐T cells had elevated expression of IFN‐γ, GzmB, and Perforin. Thus, RARα‐deficient CAR‐T cells demonstrated enhanced tumor infiltration, cytotoxicity, and TME‐modifying properties.

### 2.7 Co‐Binding of RARα and BATF on Many Teff Genes

**Figure 8:**
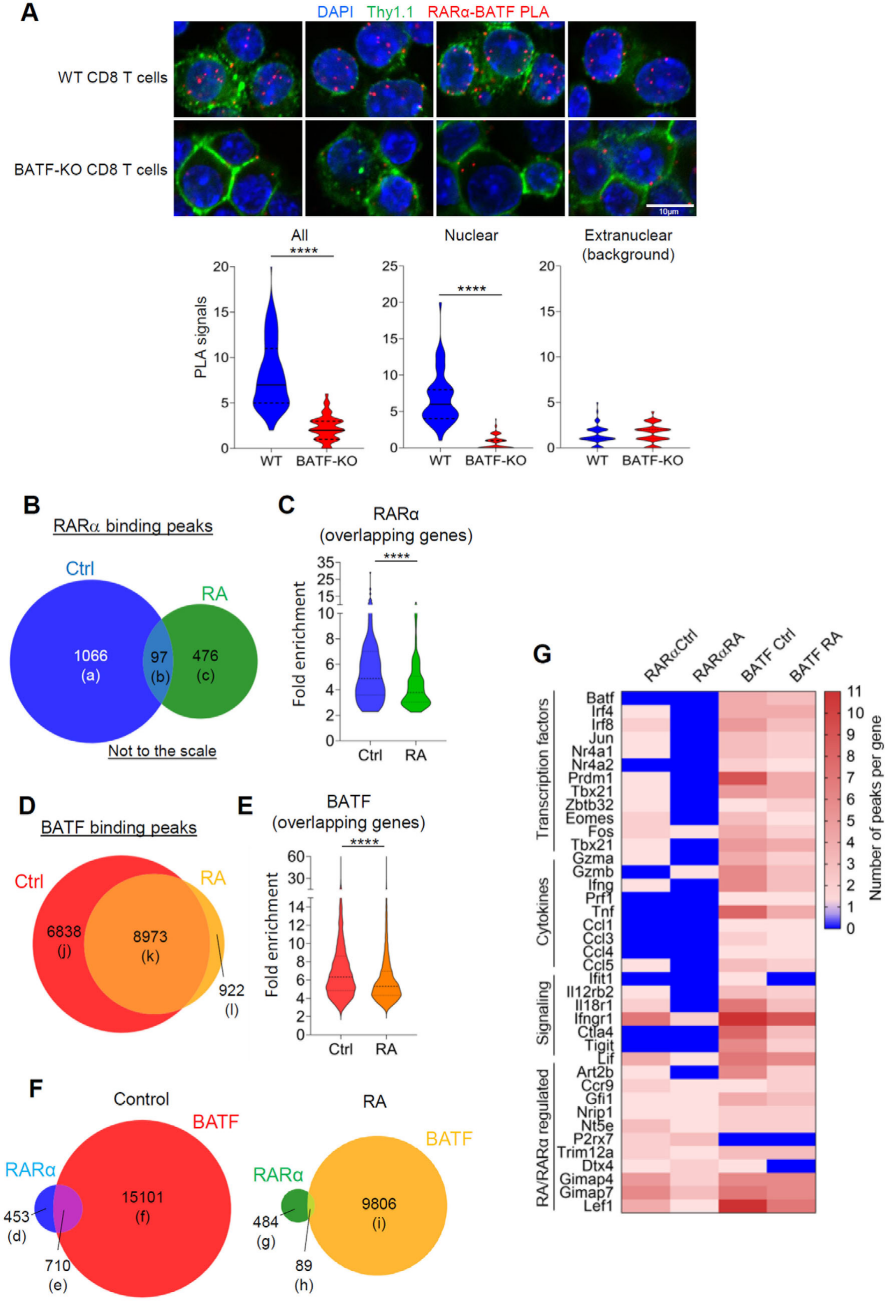
Physical and functional associations between RARα and BATF on the chromatin of CTLs. (A) Proximity ligation assay (PLA) signals for BATF and RARα in retrovirally transduced CD8 T cells. CD8 T cells were transfected with FLAG‐tagged RARα and cultured for 12 h with a physiological level of RA (15 nm) for the assay. Scale bar, 10 µm. (B) Venn diagram showing the number of RARα peaks for control (Ctrl) and RA‐treated CTLs. (C) Fold enrichment for the overlapping RARα peaks for control and RA‐treated CTLs. (D) A Venn diagram showing the number of BATF peaks for Ctrl and RA‐treated CTLs. (E) Fold enrichment for the overlapping BATF peaks for control and RA‐treated CTLs. (F) Impact of RA on RARα and/or BATF binding peaks. (G) Representative peaks with the binding of RARα and/or BATF. (H) Impact of RA on the number of AP‐1 motif‐containing target sequences. For all ChIP‐seq experiments, 24 h‐preactivated CTLs were cultured for 1 h with or without RA (50 nm). Statistical significance was determined using two‐tailed, unpaired *t*‐test (A) or two‐tailed paired *t*‐test (C,E). ^*^ *p* ≤ 0.05; ^****^ *p* ≤ 0.0001.

RARα transactivates genes with retinoic acid elements (RAREs).^[^ ^5^ ^]^ The products of transactivation include *Ccr9, Itga4, P2rx7, Nt5e*, and others, but the functions of these genes hardly affect Teff differentiation. Another function potentially important for the regulation of Teff genes is the suppression of AP‐1 proteins by RARα.^[^ ^31^ ^]^ Because BATF is an AP‐1 family protein, the negative regulation by RARα may well be extended to BATF. To determine the potential interaction between RARα and BATF, we performed a proximity ligation assay (PLA), which detects proximity between molecules at <40 nm resolution. We detected PLA signals between tagged RARα and natural BATF in CTLs, which were largely absent in BATF‐KO CTLs (**Figure** **8**A). We performed a protein immunoprecipitation assay for the nuclear proteins of similarly prepared CTLs with anti‐RARα but did not find BATF‐RARα interaction in solution (not shown). This suggests that the interaction is too weak to detect in solution and/or limited to chromatin‐bound molecules in CTLs.

To assess the binding pattern of the two factors on the chromatin, we performed chromatin immunoprecipitation sequencing (ChIP‐seq) for CTLs activated in RA‐depleted and replete conditions. We found 1163 binding sites for RARα and, interestingly, this number decreased by ∼50% in the presence of RA (Figure 8B,C). Unexpectedly, the binding sites for RARα in RA‐depleted and replete conditions were largely distinct, suggesting that RA changed the binding specificity of RARα. RA also had a modest negative effect on the total number of BATF‐binding peaks (Figure 8D,E). The RA‐induced decrease in RARα binding was confirmed on several RA‐induced genes, such as *Lif, Gfi1*, and *Asb2* (Figure S15A,B, Supporting Information). Surprisingly, the majority (∼61%) of RARα‐binding chromatin regions had BATF co‐binding activity in the RA‐depleted condition (Figure 8F,G; Figure S15C, Supporting Information), which included *Lef1, Ccl5*, and *Lig1*. In contrast, genes such as *Art2b, Ncoa3* and *Deptor* had largely RARα binding only, whereas genes such as *Tbx21, Stat1*, and *Mki67* had mainly BATF binding only. In the presence of RA, however, most of the co‐binding activity was eliminated, suggesting that RA affects the binding of RARα on the chromatin (Figure 8F,G). Importantly, some of the BATF‐binding loci are also occupied by RARα, and these chromatin loci included Teff transcription factors (*Irf4, Irf8, Prdm1, Jun, Nr4a1*, and *Tbx21*) and effector molecules/cytokines/receptors/chemokines (*Gzma, Ifng, Ccl5, IL12rb2, Il18r1*, and *Ifngr1)* (Figure 8G). Overall, the results identified co‐binding of BATF and RARα to important effector gene loci in CTLs, and that this co‐binding is surprisingly downregulated by its ligand RA. This regulation is expected to have significant effects on Teff differentiation.

### 2.8 Major Teff Genes in CTLs are Up‐Regulated by both RARα Deficiency and RA in a BATF‐Dependent Manner

**Figure 9:**
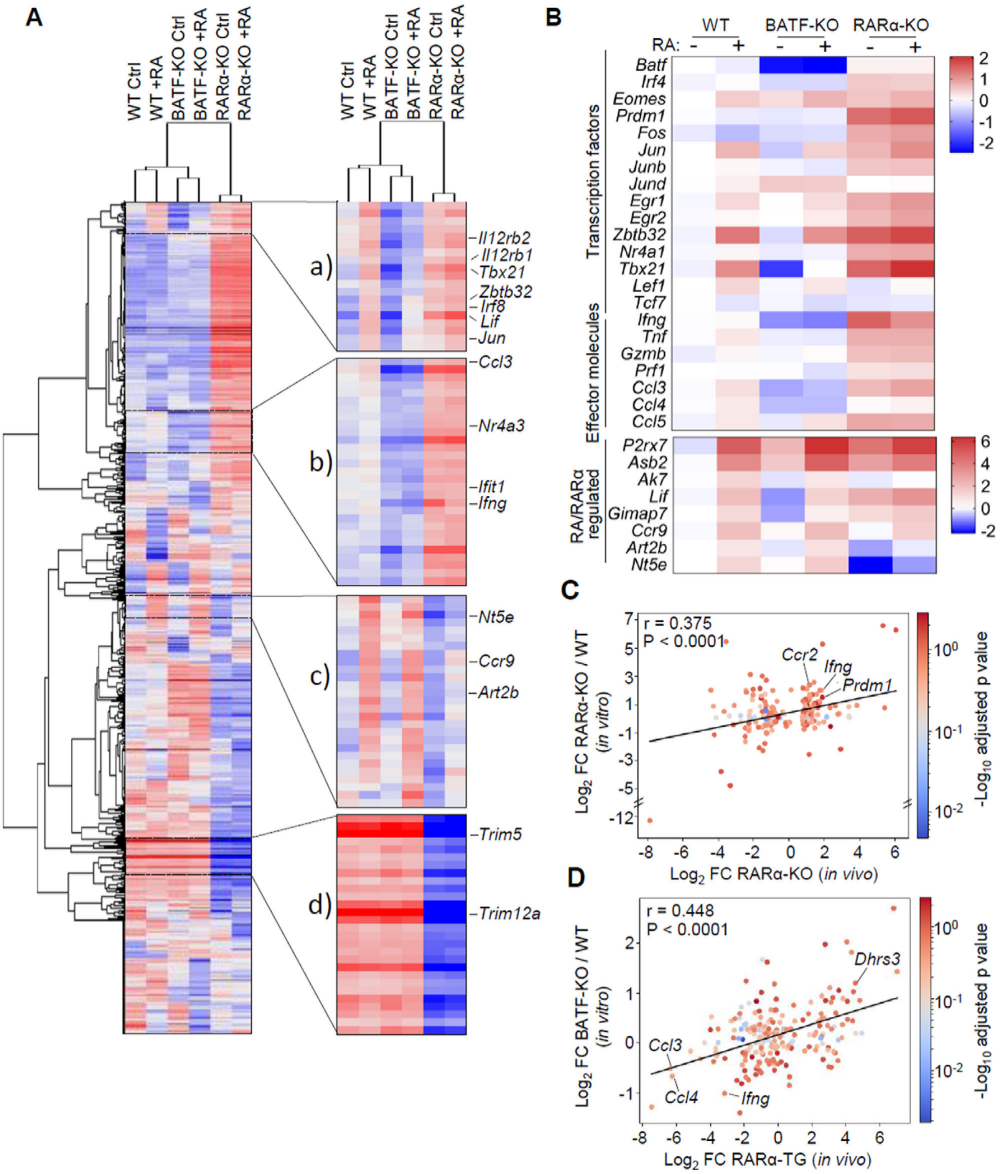
RARα deficiency and RA commonly increase the expression of many BATF‐regulated genes important for Teff functions. (A) Heatmaps showing DEGs among activated naïve WT, RARα‐KO, and BATF‐KO CTLs treated for 20 h with or without RA. (B) Selected genes of interest regulated by RA, BATF, and/or RARα. (C) The correlation between the RARα‐regulated genes in vitro versus in vivo within tumors. The log2 fold‐change in the expression of DEGs by RARα‐KO *Cd8a* ^+^ T cells (in vivo scRNA seq data) was plotted against the log2 fold‐change of corresponding genes from RARα‐KO versus WT *Cd8a* ^+^ T cells (in vitro bulk RNA seq data). (D) The correlation between the RARα and the BATF‐regulated genes. The log2 fold‐change in the expression of DEGs in RARα‐TG *Cd8a* ^+^ T cells (in vivo scRNA seq data) was plotted against the log2 fold‐change in the expression of corresponding genes from BATF‐KO versus WT *Cd8a* ^+^ T cells (in vitro bulk RNA seq data). The color intensity of dots indicates ‐log10 adjusted *P* value for bulk RNA seq. The values for Pearson correlation coefficient (r) and significance (*P*) are shown.

To further understand the impact of RARα and BATF binding to the chromatin on gene transcription, we performed bulk RNA‐seq analysis of 20 h‐cultured WT, BATF‐KO, and RARα‐KO CTLs (**Figure** **9**A). We found multiple groups of gene clusters that were regulated differently by RARα, RA, and BATF. The genes in group (a) and (b) that were increased in their expression in RARα deficiency included *IL12rb1, IL12rb2, Tbx21, Zbtb32, Irf8, Lif, Jun, Ccl3, NR4a3, Ifit1* and *Ifng*, most of which are required for Teff development. Many of these genes, particularly those in group (a), were also increased in expression in WT CTLs by RA. Thus, RA partially mimics the effect of RARα deficiency in the expression of key effector‐related genes. However, these genes were largely inactive and not regulated in BATF‐KO CTLs by RA, suggesting that BATF is required for the induction of these genes by RA or RARα deficiency. In contrast, RA induces the expression of group (c) genes which included the classic RARE‐regulated genes, such as *Nt5e, Ccr9*, and *Art2b*, and this induction was largely abolished in RARα deficiency as expected. Interestingly, BATF deficiency did not affect the expression of these genes in CTLs. Many genes were downregulated in RARα deficiency, but RA had no effect on these genes in group (d). The expression of *Batf, Irf4, Prdm1, Fos, Jun, Junb, Egr1, Egr2*, and *Tbx21* was increased in RARα‐KO CTLs, and some of these transcription factors were up‐regulated by RA (Figure 9B). *Tcf7*, which encodes TCF1, expressed by naïve and effector precursor but not effector/exhausted CTLs,^[^ ^32^ ^]^ was down regulated by both RA and RARα deficiency (Figure 9B). In contrast, the expression of key effector molecules and chemokines that attract Teff cells into tumors^[^ ^22^, ^33^ ^]^ was increased by both RA and RARα deficiency in a BATF‐dependent manner.

To establish the validity of the bulk RNA‐seq data obtained for in vitro‐activated CTLs as a model of gene expression in tumor‐infiltrating CTLs, we additionally examined the relationship between the in vitro bulk‐RNA‐seq data and the in vivo tumor‐infiltrating lymphocyte (TIL) scRNA‐seq data. We found a positive correlation between the genes upregulated in RARα‐KO tumor‐infiltrating CTLs and the genes upregulated in vitro in RARα‐KO versus WT CTLs (Figure 9C). Moreover, there is a significant positive correlation between BATF‐regulated genes (i.e., DEGs between BATF‐KO versus WT CTLs) and the RARα‐regulated genes (i.e., DEGs between RARα‐TG versus WT tumor‐infiltrating CTLs) (Figure 9D). This information indicates that RARα exerts a suppressive effect on the expression of BATF‐induced genes.

### 2.9 RARα Negatively Affects HAT Activity, Which is Important for both the TCF1‐BATF and Trafficking Receptor Switches in Activated CTLs

**Figure 10:**
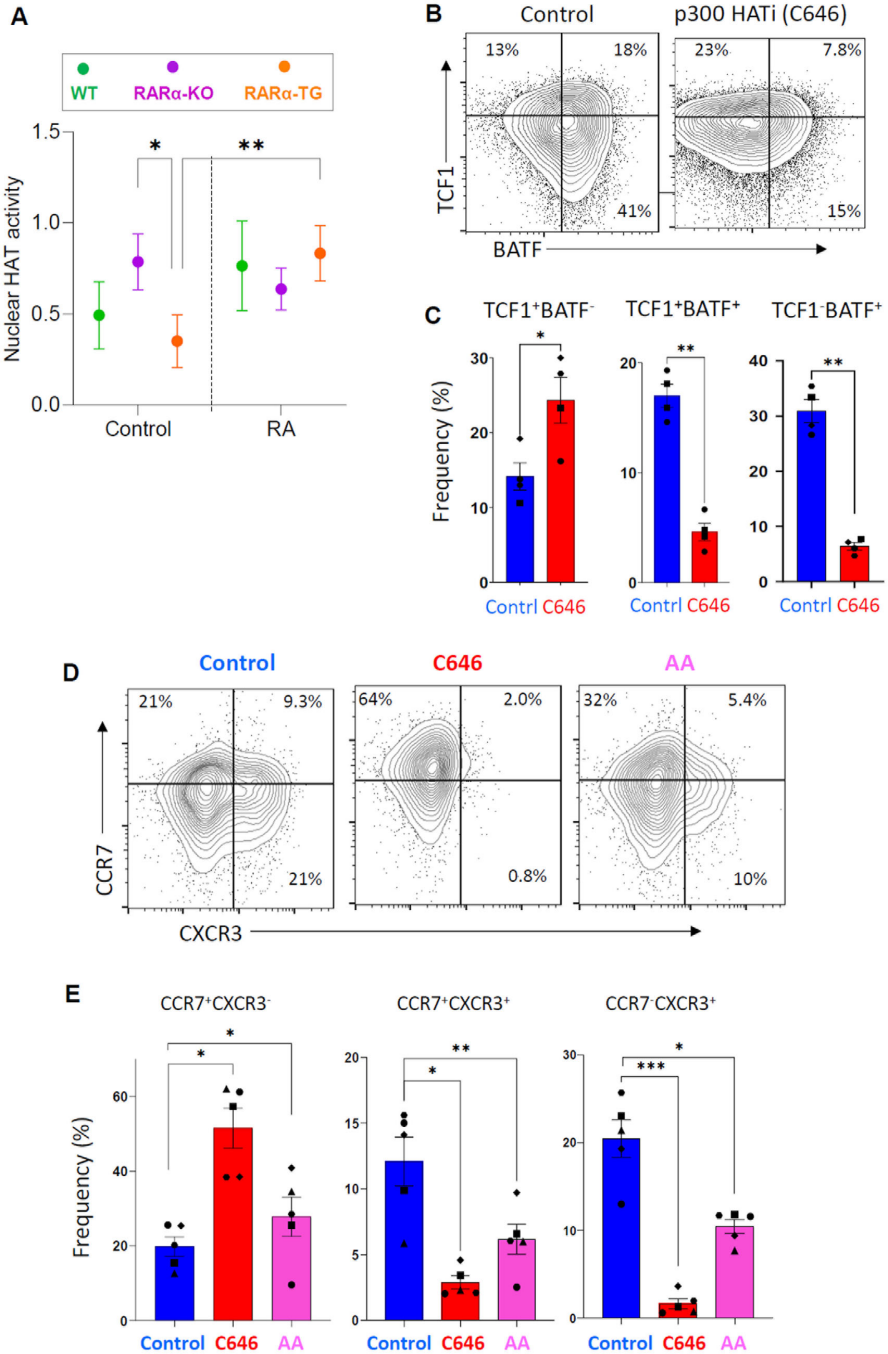
The nuclear HAT activity, critical for transcriptional factor and trafficking receptor switches, is regulated by RARα in CTLs. (A) Total HAT activity in CTLs from WT, RARα‐KO, or RARα‐TG mice. (B,C) Impact of p300 HATi (C646, 5 µm) on TCF1 and BATF expression. Frequencies of TCF1^+^ BATF^−^, TCF1^+^ BATF^+^, and TCF1^−^ BATF^+^ CTLs from cultured OT‐1 T cells. (D,E) Impact of HATi (C646 and anacardic acid/AA) on the CCR7 to CXCR3 switch. Frequencies of CCR7^+^ CXCR3^−^, CCR7^+^ CXCR3^+^, and CCR7^−^ CXCR3^+^ CTLs were examined in cultured OT‐1 T cells. For panel B‐E, OT‐1 T cells, cultured for 5 days with repeated stimulation with the OVA SIINFEKL antigen peptide in the presence of IL‐7 and IL‐15, were examined. Statistical significance was determined using two‐way ANOVA with Tukey's multiple comparisons test (*n* = 4 for panel A) and Student's paired *t*‐test in panel (C,E); ^*^ *p* ≤ 0.05; ^**^ *p* ≤ 0.01; ^***^ *p* ≤ 0.001.

RARα recruits HATs, such as p300/CBP, for gene expression.^[^ ^8^ ^]^ We measured the total nuclear HAT activity in 20h‐stimulated CTLs from WT, RARα‐KO, and RARα‐TG mice, and found high and low nuclear HAT activity, respectively, in RARα‐KO and RARα‐TG CTLs (**Figure** **10**A). This suggests that RARα decreases the nuclear HAT activity. Interestingly, RA has a tendency of increasing the HAT activity in WT and RARα‐TG CTLs but not in RARα‐KO CTLs. We utilized a p300 HAT‐specific inhibitor (C646) for the OVA‐specific OT‐1 CTL culture system with repeated stimulation with the OVA_257‐264_ peptide to mimic TME to determine if p300 HAT is required for BATF up‐regulation, which was found to be differentially regulated in RARα‐KO and RARα‐TG CTLs in tumors (Figure 6E). We found that C646 increased TCF1 expression and the generation of less differentiated early‐stage TCF1^+^BATF^−^ CTLs (mirroring the RARα‐TG CTLs in tumors) but suppressed BATF expression and the generation of more differentiated TCF1^−^BATF^+^ cells (mirroring the phenotype of RARα‐KO CTLs in tumors) (Figure 10B,C).

Moreover, p300 HAT inhibition with C646 increased CCR7‐expressing but suppressed CXCR3‐expressing OT‐1 T cells after repeated stimulation with the cognate OVA_257‐264_ peptide for 5 days (Figure 10D,E). Anacardic acid (AA), another inhibitor of the co‐activators p300 and p300/CREB‐binding protein‐associated factor, had similar effects as C646 on the trafficking receptor switch. A similar effect of the HAT inhibitors was observed on 2‐day‐cultured OT‐1 T cells with acute stimulation (Figure S16A,B, Supporting Information). However, HDAC inhibition by Trichostatin A had no or only a small positive effect on the expression of BATF and CXCR3 (not shown). Thus, the data indicates that the p300 HAT activity, regulated by RARα expression, is involved in the transcriptional factor and Teff trafficking receptor switches in primed CTLs.

## 3 Discussion

The function of RARα in regulating Teff CTL differentiation in TME has been unclear. The recent findings in the field on the negative function of several nuclear receptors in regulating CTLs called for an in‐depth examination of the intrinsic function of RARα.^[^ ^4^ ^]^ Our study revealed a novel negative function of RARα during anti‐tumor Teff CTL differentiation. We found that RARα expression is upregulated as CTLs differentiate from naïve to Teff and to Tex‐like cells in tumors. We investigated the roles of increased or decreased expression of RARα on anti‐tumor CTLs by utilizing animal models with altered expression of RARα. Overall, we found that RARα negatively regulates Teff differentiation, including the changes in transcriptional factors, effector molecules, and trafficking receptors in TME (Figure S17, Supporting Information). We also demonstrated that the outcomes of this study can be utilized to make a potentially effective CAR T therapy by reducing RARα expression. The results establish a novel function of RARα in negatively regulating cytotoxic T cells in TME.

The negative function of RARα reported in this study is reminiscent of those of other nuclear receptors, such as NR4A1, AR, GR, and LXRβ.^[^ ^4^ ^]^ NRs share similar protein structures such as transactivation regions (A/B), a conserved DNA‐binding domain (C), nuclear localization domain (D), and a conserved but distinct carboxy‐terminal ligand‐binding domain (E).^[^ ^7^ ^]^ These NRs appear to decrease AP‐1/TRE‐mediated transcriptional activation.^[^ ^34^ ^]^ In this regard, NR4A1 and NR4A3 have been reported to suppress pathways regulated by the AP‐1 family transcription factor BATF in B cells.^[^ ^35^ ^]^ We found that RARα also suppresses the expression of many genes induced by BATF.^[^ ^26a^ ^]^ Moreover, the expression of BATF itself was severely suppressed in RARα‐TG CTLs but increased in RARα‐KO CTLs in tumors. One of the major functions of RARα is to recruit HATs or HDACs depending on ligand concentrations.^[^ ^8^ ^]^ In this regard, RARα‐KO CTLs had upregulated levels of nuclear HAT activity compared to RARα‐TG CTLs, and this would promote Teff gene expression. p300 is a RARα‐associated co‐activator with a high HAT activity.^[^ ^8^ ^]^ We found that the inhibition of p300 suppressed the TCF1 to BATF transcriptional switch necessary for Teff differentiation. The negative regulation of the nuclear HAT activity by RARα also provides an effective mechanism for the decreased trafficking receptor switch and tumor infiltration in RARα‐TG mice.

In addition to transcriptional activation through TRE/AICE, BATF works with CCCTC‐binding factor to induce chromatin remodeling to facilitate Teff gene expression.^[^ ^25^, ^26^, ^36^ ^]^ Our data revealed that RARα closely associates with BATF on the chromatin in CTLs at protein level. The RARα interaction with BATF was revealed by PLA and ChIP‐seq approaches on the chromatin. The close interaction between RARα and BATF suggests that RARα has the potential to crosstalk with BATF during Teff differentiation. The ChIP‐seq study revealed that some of the BATF‐binding loci are also occupied by RARα. The genes at these chromatin loci (e.g., *Irf4, Irf8, Prdm1, Jun, Nr4a1, Tbx21*, *Gzma, Ifng, Ccl5, IL12rb2, Il18r1*, and *Ifngr1)* play key roles in Teff differentiation and function. While the functional importance remains to be investigated in CTLs, chromatin‐attached BATF was frequently found on the conventional RA‐regulated genes.

The enhanced Teff function of RARα‐deficient CTLs in tumors, observed in this study, is interesting and novel. It has been described also that RARα acutely promotes T cell activation signaling, such as activation of TCR and PI3K, potentially via its non‐genomic functions, which are suppressed by RA.^[^ ^9^ ^]^ In this regard, RARα‐deficient CTLs failed to mount normal effector functions during an acute *L. monocytogenes* (LM) infection.^[^ ^37^ ^]^ The negative function of RARα in CTLs in TME observed in his study indicates that RARα exerts distinct effects depending on the context of immune responses (e.g., chronic tumor versus acute bacterial infection), tissue environments (i.e., tumors versus spleen), cell types (CD4/CD8 or stages of differentiation) and the expression level of RARα. In this regard, the expression of *Rara* is upregulated in Teff/Tex in tumors. Further studies are required to dissect the potentially distinct functions of RARα in acute versus chronic conditions.

An interesting phenotype of RARα‐TG mice is the drastically reduced number of CTLs infiltrating tumors (Figure 2E), which could be due to trafficking problems. For optimal migration of Teff CTLs, they must undergo a normal trafficking receptor switch from lymphoid tissue to effector‐site homing receptors. In this regard, we demonstrated that the CCR7→ CXCR3 switch was significantly decreased in RARα‐TG CTLs in tumor‐draining lymph nodes. While CXCR3 was examined as the representative trafficking receptor for Teff CTLs, this can be extended to other trafficking receptors such as CCR1, CCR2, and CCR5 based on RNA‐seq data (Figures S10 and S11, Supporting Information). Chromosome unpacking for gene expression requires an optimal activity of nuclear HATs which are recruited to liganded holo‐RARα.^[^ ^38^ ^]^ We found an inverse correlation between the RARα expression level and the nuclear HAT activity. Moreover, we observed that a decreased p300 HAT activity in antigen‐primed CTLs decreased the trafficking receptor switch during Teff CTL differentiation. Thus, these results provide a potential mechanism for the observed difference in CTL infiltration into tumors of RARα‐KO versus RARα‐TG mice.

We used dLCK‐Cre or CD2 to delete or over‐express RARα expression, and these gene promoters regulate RARα expression in all T cells. In this regard, there is a possibility that the observed changes in CTL responses could be the result of indirect regulation mediated through CD4^+^ T cells. However, the CD4^+^ T cells in tumors were less affected by RARα deficiency compared to CTLs, suggesting that RARα may function differently in CD4 versus CD8 T cells. RARα promotes, rather than suppresses, early activation of CD4^+^ T cells by activating cell signaling for increased metabolism and Teff differentiation.^[^ ^9^, ^10^ ^]^ While it is controversial in vivo, RA induces Tregs in vitro.^[^ ^39^ ^]^ Our scRNA‐seq analysis indicates that increased RARα expression may lead to a modest increase in T cells expressing *Foxp3* in tumors, but we failed to validate this in tumors by flow cytometry. This is in line with our previous observation that the induction of FoxP3^+^ T cells in the periphery does not require vitamin A or RA.^[^ ^11b^ ^]^ The results of our T cell transfer study using CAR T cells further support that the negative effect of RARα on CTL is indeed a CTL‐intrinsic effect.

Our findings present potential application points. RARα deficiency led to the increased CAR‐T cell performance in a manner similar to the effect of overexpressed BATF or decreased expression of other transcription factors such as NR4A.^[^ ^4^, ^26^ ^]^ We did not find significant differences in the tumor‐killing activities between WT and RARα‐TG CAR T cells. This is probably due to the use of a suboptimal number (i.e., 1.5 million per mouse) of CAR T cells in this study to optimally detect the difference between WT and RARα‐KO CAR T cells, rather than that between WT and RARα‐TG CAR T cells. Additionally, the retroviral gene transfer process requires pre‐activation in RA‐containing medium, which can decrease the overexpressed RARα in RARα‐TG T cells. Also, the signaling modules in the CAR construct may allow RARα‐TG T cells to partially overcome the suppressive effect of RARα.

Considerable variations in the production of RAs depending on types of cancers are expected based on the heterogeneous infiltration of myeloid cells and expression of the enzymes that produce or degrade RAs in different tumors. While the 26S proteasome pathway has been identified as a pathway for the degradation of RARα in cell lines,^[^ ^18^, ^40^ ^]^ we observed in this study that two potent inhibitors of this pathway failed to decrease RA‐induced degradation of RARα in CTLs. We also observed that inhibitors of alternative protein degradation pathways, such as the lysosomal and calpain pathways, did not block the degradation either. This suggests that the RA‐induced degradation of RARα in CTLs is a highly efficient process that is not mediated by the conventional protein degradation pathways. Additional studies are required to identify the mechanism of RARα degradation in CTLs. Overall, the RA‐dependent degradation of RARα has the potential to limit the negative effect of RARα in CTLs in tumors. This also provides a potential explanation for the similarity between RARα deficiency and RA in the expression of key Teff genes in CTL cells.

## 4 Limitations

Our findings suggest the negative regulation of HAT activity (e.g., p300) and BATF by RARα as the potential mechanisms. Future studies are required to understand more detailed mechanisms by which RARα regulates HATs in CTLs. It is also important to validate the role of p300 or other HATs involved in this process. Our data suggests that BATF is a major target of the negative regulation by RARα. For the suppression of BATF expression by RARα, we found that HAT regulation by RARα is linked to the downregulation of BATF. The full validation of the connection from RARα‐regulated HATs to BATF expression requires additional studies at the molecular level. Independent of the regulation of BATF expression, we presented data that RARα and BATF interact and potentially crosstalk to each other. The functional consequence of this interaction also needs additional studies. We presented data that RA abolishes some of the effects of RARα on Teff gene and protein expression. Further research is warranted to fully understand how the negative function of RARα is modified by its ligand RA. Particularly, it is important to dissect the relative roles of RA in limiting the suppressive function versus the conventional transactivation function in CTLs. We employed two experimental tumor models for the study and found considerable variations. While the effects of RARα on the CTL phenotype in both tumor models were similar and consistent, the overall effect on tumor growth was more pronounced in MC38, compared to B16, tumors. We speculate that this would be due to the fact that MC38 tumors are more immunogenic and conducive for T cell infiltration than B16 tumors, and therefore more amenable to the RARα‐regulated CTL responses. Further studies with additional tumor models are required to delineate the reason behind the heterogeneity in tumor responses. It is particularly important to determine if this is due to potential differences in their production of RA. Also, more in‐depth studies on CTLs in human cancers would be studied in parallel to those on mouse CTLs. Finally, we did not study if the observed negative effect of RARα on Teff differentiation occurs specifically in TME or broadly in various types of infections. It remains unclear if RARα has any role in regulating T cell exhaustion in chronic conditions. These are important questions that warrant follow‐up studies with appropriate experimental models.

## 5 Conclusion

In sum, we report a novel function of RARα in limiting Teff CTL differentiation in tumors (Figure S17, Supporting Information). We found that RARα is not just a dedicated receptor to convey the RA signal but has its own function as a suppressor of the Teff gene expression program. At molecular level, upregulated RARα suppresses the Teff differentiation‐associated transcription factor expression and trafficking receptor switch. The negative regulation of Teff differentiation would be important not only for immune tolerance to suppress unwanted activation of CTLs in the steady state but also potentially presents a significant problem in mounting effective anti‐tumor CTL responses in TME. We provided evidence that RA functions to relieve some of the suppressive effect of RARα on the expression of Teff genes. These findings present novel application points in anti‐cancer and other CTL‐based therapies.

## 6 Experimental Section

### Animals

The animal protocol for the study was approved by the Animal Care and Use Committees at the University of Michigan (PRO00009958). This study utilized C57BL/6 (Taconic Biosciences) and dLck‐Cre (the Jackson laboratory, strain 012837) mice as the control WT strains and used Rag1‐KO mice (the Jackson laboratory, stock 002216) as the host for T cell transfer. CD2‐Rara (RARα‐TG) and dLck‐Cre×Rara L/L (RARα‐KO) mice were described previously.^[^ ^9a^ ^]^ Also used were C57BL/6‐Tg(TcraTcrb)1100Mjb/J (OT‐1, strain 003831 from the Jackson laboratory, stock 002216) mice. These mice were produced from breeders housed at the institution for more than 12 months. Both male and female mice were used for initial studies but later focused on male mice for most of the experiments. All mice were kept under a specific pathogen–free condition on a regular rodent chow ad libitum on the 12‐h dark and 12‐h light cycle. Most experiments were performed on age‐matched 6‐ to 10‐week‐old male and female mice.

### Cells and Cell Culture

MC38 colon adenocarcinoma cells (PTA‐5817, ATCC) and B16‐F10 murine melanoma cells expressing ovalbumin (B16) cells^[^ ^41^ ^]^ were used for the study. hCD19‐MC38 and hCD19‐B16 were kindly provided by Dr. Anjana Rao.^[^ ^4a^ ^]^ Tumor cells were thawed and cultured in Dulbecco's modified Eagle's medium supplemented with 10% fetal bovine serum (FBS, Thermo Fisher Cat# 12483020), 1% L‐glutamine and 1% penicillin/streptomycin at 37 °C in a 5% CO2 incubator. Cells were passaged once after thawing before implantation in mice. Platinum‐E packaging cells were cultured in Dulbecco's modified Eagle's medium supplemented with 10% FBS, 1% L‐glutamine, and 1% penicillin/streptomycin at 37 °C in a 5% CO2 incubator.

Naïve CD8 T cells with a purity between 90 and 98% were isolated from spleens using the Naïve CD8 T cell isolation kit using the AutoMACS system (Miltenyi Biotech). CD8 T cells were cultured on 96 or 48‐well plates coated with anti‐CD3 (Bio X Cell clone 145‐2C11, 1 µg mL^−1^) in RPMI supplemented with anti‐CD28 (Bio X Cell clone 37.51, 2 µg mL^−1^) and human IL‐2 (BioLegend Cat# 589102, 100 U mL^−1^). Either 10% FBS or 10% charcoal‐stripped FBS were used for the medium. OT‐1 T cells were similarly isolated from the spleen and cultured at 150 000 cells per well (48‐well plates) in the presence of the OVA peptide (SIINFEKL, 10 ng mL^−1^) and cytokines (IL‐7 and IL‐15 at 20 ng mL^−1^) for 2 days for an acute condition. For chronic conditions, the 2‐day‐activated OT‐1 cells were repeatedly activated on days 2, 3, and 4, and the cultures were terminated on day 5 for flow cytometry analysis. When indicated, anacardic acid and C646 (Cayman Chemical) were used at an optimal concentration (5 µm) after preliminary titration experiments. When indicated, R‐MG132 (20 µm), S‐MG132 (20 µm), chloroquine (150 µm), and calpain inhibitor II (150 µm) were used at their optimal concentrations that exceeded >5x their established inhibitory concentrations (IC_50_).

### Nuclear HAT Assay for CTLs

Isolated naïve spleen CD8 T cells from control, RARα‐KO, or RARα‐TG mice were cultured as described above with plate‐bound anti‐CD3 (1 µg mL^−1^), soluble anti‐CD28 (2 µg mL^−1^) and IL‐2 (20 ng mL^−1^) for 20 h in RPMI1640 supplemented with 10% charcoal‐treated FBS. RA was added at 20 nm. Nuclear lysates were prepared after cell membrane lysis and total HAT activity was measured using EpiQuik HAT Activity/Inhibition Assay Kit (Epigentek, Cat# P‐4003‐96).

### Adoptive T‐Cell Transfer into Rag1‐KO Mice Bearing Tumors

Rag1‐KO mice were injected subcutaneously with 2 × 10^5^ MC38 cells. After 24 h, total T cells were isolated from spleens of WT, RARα‐KO, and RARα‐TG mice using biotinylated antibodies against CD19 (clone 6D5), B220 (clone RA3‐6B2), CD11b (clone M1/70), CD11c (clone N418), Ly6G (clone 1A8) and MHC2 (clone M5/114.15.2) from BioLegend, followed by an incubation with anti‐biotin magnetic beads (Miltenyi Biotech) and negative selection of total T cells using the AutoMACS system. 2 × 10^5^ T cells with a purity greater than 92% based on CD3 expression were injected per mouse via the retro‐orbital sinus. Tumors were measured every 2–3 days for up to 28 days.

### Tumor Growth and Animal Survival

Mice were injected with 2 × 10^5^ of MC38, B16, or B16‐hCD19 cells subcutaneously into the right shaved flank. Tumors were measured every 2–3 days for at least 20 days if not otherwise indicated. For rechallenge experiments, animals with complete remission (no measurable tumor) were reinjected with 2 × 10^5^ MC38 cells 8–10 weeks after the first injection. Animals were sacrificed and considered dead once the tumor reached a volume of >1000 mm^3^, and these data were plotted as Kaplan–Meier survival curves.

### Flow Cytometry of Immune Cells

To obtain tumor‐infiltrating cells, excised tumor tissues were cut into small pieces and digested with Collagenase Type 3 (1.5 mg mL^−1^; Worthington, Lakewood, NJ) for 60 mins at 37 °C in RPMI medium supplemented with 10% newborn calf serum and 1% penicillin/streptomycin with gentle rotation. The cell suspension was passed through nylon meshes (100 µm, Fisher) and washed in phosphate‐buffered saline (PBS, pH 7.3). Agilent NovoCyte flow cytometers were used for acquisition. Single‐cell suspensions from tumors or inguinal lymph nodes were stained with antibodies for 30 m in MACS buffer (PBS with 0.5% BSA and 2 mm EDTA) on ice before acquisition. For intracellular staining of cytokines, cells were stained for surface markers, followed by activation with phorbol 12‐myristate 13‐acetate (PMA; 50 ng mL^−1^; Sigma Aldrich P8139), ionomycin (1 µg mL^−1^; Sigma Aldrich I9657) and monensin (1.5 µm; Sigma Aldrich M5273) for 4 h. Cells were fixed with 1% paraformaldehyde in PBS for at least 2 h, permeabilized with permeabilization buffer (0.1% saponin in PBS), and then stained for 1h in the same buffer with anti‐cytokine antibodies at room temperature (RT). For staining of transcription factors, cells were stained for surface markers, and fixed with the Fix/Perm reagent (Tonbo Bioscience Cat# TNB‐1022‐L160) for 15 m at RT, followed by permeabilization with 1× Perm buffer (Tonbo Bioscience) for 5 m at RT and stained for 1 h with antibodies at RT. The antibodies used for flow cytometry were anti‐mouse RARα Cell Signaling Technology Cat# 62294, RRID:AB_2799625), Alexa Flour 594 anti‐rabbit IgG (Cell Signaling Technology Cat# 8889, RRID:AB_2716249), FITC anti‐mouse CD8a (BioLegend Cat# 100706, RRID:AB_AB_312745), APC/Cyanine7 anti‐mouse CD8a (BioLegend Cat. # 100714; RRID:AB_312753), Brilliant Violet 785™ anti‐mouse CD4 (BioLegend Cat# 100453, RRID:AB_2565843), Brilliant Violet 510 anti‐mouse CD45.2 (BioLegend Cat# 109838, RRID:AB_2650900), APC anti‐mouse Ki‐67 (BioLegend Cat# 652406, RRID:AB_2561930), Anti‐mouse/human BATF (Cell Signaling Technology Cat# 8638, RRID:AB_11141425), APC anti‐mouse CD3 (BioLegend Cat# 100236, RRID:AB_2561456), ANTI‐FLAG (Sigma Aldrich Cat# F1804, RRID:AB_262044), FITC anti‐rat CD90/mouse CD90.1 (BioLegend Cat# 202503, RRID:AB_314014), APC anti‐mouse IFNγ (BioLegend Cat# 505810, RRID:AB_315404), PE anti‐mouse CCL5 (BioLegend Cat# 149104, RRID:AB_2564406), APC anti‐human/mouse Granzyme B (BioLegend Cat# 372204, RRID:AB_2687028), PE anti‐mouse Perforin (BioLegend Cat# 154406, RRID:AB_2721641), FITC anti‐mouse/human CD44 (BioLegend Cat# 103022, RRID:AB_493685), PE anti‐IRF4 (BioLegend Cat# 646404, RRID:AB_2563005), BV421 Mouse Anti‐TCF‐7/TCF‐1 (BD Biosciences Cat# 566692, RRID:AB_2869822), PE anti‐mouse FoxP3 (BioLegend Cat# 126404, RRID:AB_1089117), BV421‐anti‐mouse CD197 (CCR7, AB_10897811), and APC anti‐mouse CD183 (CXCR3, AB_1088993).

### Single‐Cell and Bulk RNA‐Sequencing and Bioinformatics Analyses

WT, RARα‐KO, or RARα‐TG mice bearing MC38 tumors were sacrificed on day 16 post tumor cell‐injection, and tumor cell suspensions were stained, respectively, with anti‐mouse TotalSeq™‐C0301, C0302, and C0303 Hashtag antibodies (BioLegend Cat# 155861/3/5, RRID:AB_AB_2800693/4/5) together with fluorochrome‐labeled antibodies against CD45.2, CD3, CD4, and CD8 for cell sorting. 30000 T cells (CD45.2^+^ CD3^+^) were sorted by FACS Melody (BD Biosciences) from total tumor single‐cell suspensions and then spiked with 10000 unsorted cells from the same tumor single‐cell suspensions for scRNA‐seq. This was to examine T cells in detail but also to examine non‐T cells in the same tumors. The cells from WT, RARα‐KO, and RARα‐TG tumors were differentially labeled with the three TotalSeq antibodies and then combined for multiplexed single‐cell RNA and TCR repertoire sequencing by 10X genomics Chromium Single Cell 5′ Reagent Chromium Single Cell Mouse TCR Amplification Kits. The library was sequenced with a NovaSeq S4 (300 cycle). Total of 6860 cells with 51 167 median reads per cell and 2284 median UMI per cell were captured. Mapping rate to the genome (mm10) was 87.23%. General gene expression was analyzed using the Loupe Browser (10x Genomics). Raw Fastq sequencing files were demultiplexed and analyzed with 10X Genomics Cell Ranger (6.1.0) software using the standard default settings and the cell ranger count command to generate quality control metrics and Cloupe files. Cell type‐specific marker genes and tSNE clustering were used to identify immune cell subsets. For example, CD8 T cells were identified based on the expression of *Cd8a* within the T cell cluster. The log2 fold‐change of significantly differentially expressed genes with an average count of >0.1 were analyzed. *p*‐values were adjusted using the Benjamin‐Hochberg correction for multiple tests. Differences in TCR genes were omitted from the analysis. TCR clonotypes (CDR3 region) were analyzed using the Loupe VDJ Browser 4.0 (10x Genomics). For trajectory analysis, the Monocle3 package was used.^[^ ^42^ ^]^ *Cd8a* ^+^ T cell clusters were subsetted and re‐clustered via Louvain clustering using the top 40 principal components. The naïve‐like cluster was chosen as the root of trajectory. Monocle3 ordered cells along a learned trajectory based on the transcriptional progress. Default parameters in Monocle 3 were used for analysis. CellChat was used for predicting cellular interactions using the CellChatDB database.^[^ ^21^ ^]^ Single‐cell RNA seq data including the expression of ligands and receptors were used to predict cell‐cell interaction networks. Selected significant interactions were displayed as chord diagrams whereby the thickness of the line illustrates the strengths of the predicted interaction. The full codes for Monocle 3 and CellChat analyses are found on GitHub (https://github.com/pniekamp113/Niekamp_et_al_2025).

For bulk RNA sequencing, CD8 T cells were cultured on anti‐CD3‐coated (1µg mL^−1^) six‐well plates in RPMI medium supplemented with 10% charcoal‐stripped FBS, anti‐CD28 (2 µg mL^−1^), and IL‐2 (100 U mL^−1^) for 20 h with or without RA (all‐trans‐Retinoic acid at 15 nm, Sigma Aldrich R2625). RNA was isolated using the RNAeasy kit (Qiagen #74104) according to manufacturers’ instructions and processed for poly‐A‐enrichment‐based library preparation for paired‐end 150 bp‐sequencing (300 cycle) in a NovaSeq S4 Shared Flowcell at the University of Michigan Advanced Genomics core. Duplicated samples were prepared for each group. BCL Convert Conversion Software v4.0 (Illumina) was used to generate de‐multiplexed Fastq files. In general, Snakemake was used to manage the bioinformatics workflow.^[^ ^43^ ^]^ The reads were trimmed, examined for quality of data, and screened for various types of contamination. Reads were mapped to the reference genome GRCm38 (ENSEMBL) and assigned count estimates to genes with RSEM v1.3.3 using STAR v2.7.8a.^[^ ^44^ ^]^ Hierarchical clusters were generated using the Gene Cluster software (3.0) with a cut‐off of ≥1.5 log2 fold‐change and data was presented using the Java TreeView software. The RNA‐seq data were deposited in NCBI Gene Expression Omnibus (GEO) (GSE254923 and GSE254925). The publicly available scRNA seq reference atlas for TILs (https://github.com/carmonalab/ProjecTILs) was loaded into R, the clusters annotated as CD8 T cells were subsetted, the DEGs between clusters were calculated, and log2‐fold changes for selected genes were plotted in heatmaps.

The patient survival data for high and low *RARA* expression for colon and gastric cancer patients were retrieved using the KM plotter (https://kmplot.com/analysis/). For *RARA* expression, the data for tumor‐infiltrating *CD8^+^* proliferating (Teff/prolif) non‐Tex and *CD8* ^+^ exhausted (Tex) cells from 38 publicly available datasets were retrieved from the Tumor Immune Single‐cell Hub 2 (TISCH2).^[^ ^15^ ^]^

### Chromatin Immunoprecipitation Sequencing

CD8 T cells, activated for 24 h with anti‐CD3, anti‐CD28, and IL‐2 and then rested for 24 h in IL‐2 (100 U/ml), were further treated with or without RA (50 nm) for 1 h. The cultured cells were fixed with 1% formaldehyde and processed with the SimpleChIP Enzymatic Chromatin IP Kit (Cell Signaling Technology CST #9003) as previously described.^[^ ^11c^ ^]^ The chromatin fragments were incubated with anti‐RARα (CST #62294), anti‐BATF (CST #8638), or Normal Rabbit IgG (CST #2729) overnight at 4 °C with rotation. The DNA samples were sent for library preparation and subjected to 151bp paired‐end sequencing according to the manufacturer's protocol (Illumina NovaSeq). Fastq files were processed for QC, trimming, and aligned by Bowtie2 to obtain Bam files and to generate BigWig files. Motif analysis (MACS v2, Homer) was performed at P values of 1e‐3 (RARα) or 1e‐5 (BATF) to identify ChIP‐Seq peaks and transcription binding motifs. R‐based differential expression package (DESeq2) was used. Normalized reads were aligned to the M. musculus genome using the Integrated Genome Browser (Version 9.1.10) and representative peaks are shown. To identify overlapping binding sites, peak locations were compared between the different groups based on the start and end location of each peak plus a tolerance of ± 5kb to account for small peak shifts and varying peak widths. Venn diagrams of overlapping genes were produced using the matplotlib module in Python (Version 3.7). Additionally, the fold enrichments for all overlapping genes were compared and plotted. The codes are deposited at GitHub (https://github.com/pniekamp113/Niekamp_et_al_2025). The ChIP‐seq data were deposited in NCBI GEO as GSE254924.

### Correlation Analyses for ChIP Seq, Bulk RNA Seq, and/or scRNA Seq Data

To identify correlations between gene regulation patterns in the genome‐wide sequencing data, gene expression changes (log2 fold‐change or ‐log10 adjusted *P* values from RNA‐seq data) and/or ChIP‐seq peak fold enrichment values were plotted against each other for corresponding genes from different experiments. The data were processed with the Pandas module and structured with customized Python classes (version 3.7). The data were analyzed and visualized using the modules: scipy, numpy, and matplotlib. The link for the codes on Github is described above. In addition to our own data, two publicly available data sets (GSE154747 and GSE192390)^[^ ^26^ ^]^ were used to compare gene expression patterns of BATF‐overexpressing or BATF‐KO CD8 T cells with gene expression patterns observed in our scRNA seq analysis. Correlation and significance were calculated using GraphPad Prism (Version 8.0).

### Production and Application of CAR‐T Cells

For production of retroviral particles, Platinum‐E cells were co‐transfected with pCL‐Eco (2 µg, Addgene #12371) and MSCV‐ RARα‐FLAG‐Thy1.1 or MSCV‐myc‐CAR‐2A‐Thy1.1 (5 µg, Addgene #127890) with Lipofectamine 3000 (Thermo Fisher Cat# L3000001) according to manufacturer's instruction. The culture medium was changed after 7 h post transfection and viral particles in culture supernatant were harvested 36 h later. For generation of transduced CD8 T cells, 24 h‐activated CD8 T cells were spin‐infected with retroviral particles in a medium supplemented with polybrene (8 µg mL^−1^) by centrifugation at 3200rpmi at 32 °C for 90 min. The infected cells were rested for 1 h at 37 °C and cultured in RPMI medium supplemented with IL‐2 (100 U mL^−1^) and FBS (10%). The retroviral transduction was repeated the next day and rested for 24 h and examined for viability and transduction efficiency. CAR‐T cells were at least 98% pure based on CD8 expression with a minimum viability of 80%. For in vivo CAR‐T cell experiments, mice were injected with hCD19‐expressing B16 cells (2 × 10^5^) subcutaneously into the right shaved flank. On day 7 post‐tumor injection, 1.5 × 10^6^ WT, RARα‐KO, or RARα‐TG CAR‐T cells were injected into the retro‐orbital sinus. Tumors were measured every 2–3 days for ≈25 days.

### Proximity Ligation Assay

CD8 T cells were transduced with ecotropic retrovirus particles harboring the MSCV‐RARα‐FLAG‐Thy1.1 construct as previously described.^[^ ^9a^ ^]^ These T cells were cultured for 12 h in RPMI medium supplemented with 10% charcoal‐stripped FBS and supplemented with RA (15 nm). The transduced T cells were stained with anti‐Thy1.1, spun onto a glass slide, fixed, and permeabilized. Proximity ligation assay (PLA) was performed according to the manufacturer's instruction (Sigma–Aldrich, DUO92101). In brief, fixed cells were stained with primary antibody staining against BATF (Cell Signaling Technology, CST #8638) and FLAG (Sigma–Aldrich, F1804). The cells were further treated with anti‐rabbit PLUS probe and anti‐mouse MINUS probe, followed by the ligation and amplification steps. The slides were stained with DAPI (Sigma–Aldrich, DUO82040) and imaged using an inverted Nikon A1 confocal microscope.

### Real‐Time Cytotoxicity Assay

hCD19‐expressing MC38 or hCD19‐expressing B16 cells (1.5 × 10^4^ per well) were seeded on 96‐well plates. After 24 h, cells were moved to an IncuCyte S3 Live Cell Analysis System (Sartorius) and imaged at 10× magnification. Next, CAR‐T cells were added at the indicated ratios, assuming that there were 1.5 × 10^4^ MC38 cells per well at the time of adding T cells. The Caspase‐3/7 reagent (2.5 µm, Essen Bioscience Cat# 4440) was added to visualize dying cells. Images were acquired every hour for 24–40 h. Data was analyzed using the IncuCyte analysis software to detect and quantify the number of green (apoptotic) cells per image. A size threshold of 150 µm2 was applied to exclude dead T cells.

### Statistical Analysis

*p*‐values were calculated using paired and unpaired two‐tailed Student's *t*‐tests or one‐way and two‐way ANOVA tests with Tukey's multiple comparison or Honest Significant Difference (HSD) test with GraphPad Prism (version 10.2.0). Data are shown as means ± SEM. Sample size for each experiment is shown in figure legends. Significances for Kaplan–Meier survival curves were calculated with a log‐rank (Mantel‐Cox) test with degree of freedom of 1. At least three independent experiments were performed unless otherwise indicated. *p* < 0.05 was considered statistically significant. ^*^ *p* < 0.05, ^**^ *p* < 0.01, ^***^ *p* < 0.001, and ^****^ *p* < 0.0001. Numerical data, statistics, and *p* values are provided in the source data file. All data values are listed in the source data file.

## Conflict of Interest

A provisional patent application related to this work has been filed.

## Author Contributions

P.N. performed most of the experiments and analyzed the sequencing data in consultation with C.K. R.H. and A.J. measured HAT activity, trafficking receptor expression, and RARα down‐regulation with inhibitors. C.K. conceived the project, provided research ideas and resources including animal models, directed the study, and obtained funding. N.K. participated in the correlation analyses of sequencing data, and R.K. participated in reading tumor growth. All participated in the production of the final version of the manuscript.

## Supporting information



Supporting Information

Supplemental Movie 1

Supplemental Movie 2

Supplemental Movie 3

Supplemental Movie 4

Supplemental Movie 5

Supplemental Movie 6

Supplemental Movie 7

Supplemental Movie 8

## Data Availability

The data that support the findings of this study are available in the supplementary material of this article.
