# Supplementary material for: The Nuclear Receptor NR1B1/RARα Arrests the Differentiation of Anti‐Tumor Effector Cytotoxic T Cells
Source: Adv Sci (Weinh). 2025 Mar 11;12(17):2410241. doi: 10.1002/advs.202410241 (PMC12061256; doi:10.1002/advs.202410241)
Supplement: Supplementary file 1 — Supporting Information [file ADVS-12-2410241-s004.docx]

**Supporting Information**

**The nuclear receptor NR1B1/RARα arrests the differentiation of anti-tumor effector cytotoxic T cells**

Patrick Niekamp, Ryun Hee Kim, Adithyan Jayaraman, Nils Klement, Raymond Kostlan, and Chang H. Kim

**Figure S1. Ligand-induced RARα downregulation is not suppressed by chloroquine, calpain inhibitor II, or MG-132 in CTLs.**

**Figure S2. Expression of RARα by CTLs in tumors.**

**Figure S3. Impact of T cell-expressed RARα on tumor growth and T cell responses.**

**Figure S4. Frequencies of T cells in tumor-bearing RARα-KO, and RARα-TG mice.**

**Figure S5. Frequencies of CTLs expressing exhaustion markers in dLNs and tumors.**

**Figure S6. FoxP3^+^ regulatory T cells in the dLNs and MC38 or B16 tumors of WT, RARα-KO, and RARα-TG mice.**

**Figure S7. Frequencies of CD62L^+^CD44^-^, CD62L^+^CD44^+^ and CD62L^-^CD44^+^ CD8 T cell subsets in the iLN following primary and secondary MC38 tumor challenges**

**Figure S8. Expression of selected genes in the CTL clusters from scRNA-seq data.**

**Figure S9. Impact of T cell-expressed RARα on the expression of effector molecules in CTLs.**

**Figure S10. A CellChat systemic bioinformatics analysis for major cellular interactions in MC38 tumors of WT, RARα-KO, or RARα-TG mice.**

**Figure S11. Expression of major trafficking receptors and effector molecules in CTLs.**

**Figure S12. Impact of T cell-expressed RARα on the transcription factors in CTLs.**

**Figure S13. T cell-expressed RARα affects the myeloid cells in tumors.**

**Figure S14. RARα-deficiency improves *in vitro* CAR T cell cytotoxicity.**

**Figure S15. Selected genes that have peaks for the binding of RARα and BATF on the CTL chromatin in RA-depleted (control) and replete (RA) conditions.**

**Figure S16. Inhibition of the p300 HAT decreases the trafficking receptor switch in CTLs.**

**Figure S17**. **A proposed negative role of RARα in regulating anti-tumor Teff CTLs.**

**Figure S1. Ligand-induced RARα downregulation is not suppressed by chloroquine, calpain inhibitor II, or MG-132 in CTLs.** Naive CD8 T cells, isolated from RAR-TG spleens, were activated with anti-CD3/28 and indicated inhibitors for 2h and then treated with RA (100 nM) for 18h. RARα expression was measured by two step staining by flow cytometry. Data are presented as means ± SEM. Statistical significance between two groups was determined by Student’s paired T-test (n=4).

**Figure S2. Expression of RARα by CTLs in tumors.** RARα expression by CTLs in B16 tumors of WT, RARα-KO, and RARα-TG mice ~20 days after tumor cell implantation. Statistical significance was determined using one-way ANOVA. Flow cytometry was performed. **P* ≤ 0.05; ***P* ≤ 0.01; ****P* ≤ 0.001; *****P* ≤ 0.0001.

**Figure S3. Impact of T cell-expressed RARα on tumor growth and T cell responses.**

(A) B16 tumor growth in WT, RARα-KO, and RARα-TG mice. (B) Frequency of mice with tumors greater than 700 mm^3^ on day 16 (n ≥ 12 mice per group). (C) MC38 tumor growth in Rag1-KO mice reconstituted with T cells from WT, RARα-KO, or RARα-TG mice. Individual (dotted lines) and average (bold) tumor growth curves are shown. The tumor size on day 20 is additionally shown with SEM. (D) The experimental design to assess the memory T cell response to MC38 tumor cells in RARα-KO mice. (E) Tumor growth on the flank of RARα-KO mice challenged with MC38 tumor cells for the first time on naïve mice or the second time on the counter lateral side 30-60 days after the previous tumor rejection. Also shown are the proportion of tumor-free mice on day 20. (F) Numbers and frequencies of tumor-infiltrating CD4 and CD8 T cells in WT, RARα-KO, or RARα-TG mice bearing B16 tumors. Data are means ± SEM. n ≥ 8 mice. Statistical significance was determined using one-way ANOVA with Tukey’s multiple comparison test (C, F). *P ≤ 0.05; **P ≤ 0.01; ****P ≤ 0.0001.

**Figure S4. Frequencies of T cells in tumor-bearing RARα-KO and RARα-TG mice.** (A, B) Frequencies of CD4 and CD8 T cells in the inguinal LN (iLN) of WT, RARα-KO, or RARα-TG mice bearing MC38 cells compared to naïve mice without tumor implantation. (C, D) Frequencies of CD4 T cell subsets in the iLN of naive and MC38 tumor-bearing mice. Statistical significance was determined using two-way ANOVA with Tukey’s multiple comparison test. *P ≤ 0.05; **P ≤ 0.01; ***P ≤ 0.001; ****P ≤ 0.0001.

**Figure S5. Frequencies of CTLs expressing exhaustion markers in dLNs and tumors.** (A) Expression of PD1 and Tim3 by CD8 T cells. (B) Expression of PD1 and TCF1 by CD8 T cells. CD8 T cells in the dLN and tumors of WT, RARα-KO, or RARα-TG mice bearing MC38 cells were examined by flow cytometry. Data are shown as means ± SEM (n =5-9).

**Figure S6. FoxP3^+^ regulatory T cells in the dLNs and MC38 or B16 tumors of WT, RARα-KO, and RARα-TG mice.** (A) Frequencies of FoxP3^+^ Tregs in iLN and tumors of MC38-tumor-bearing mice. (B) Frequencies of FoxP3^+^ Tregs in iLN and tumors of B16- tumor-bearing mice. Data are means ± SEM. n ≥ 4 mice. Statistical significance was determined using one-way. ANOVA with Tukey’s multiple comparison test. *P ≤ 0.05; **P ≤ 0.01; ***P ≤ 0.001; ****P ≤ 0.0001.

**Figure S7. Frequencies of CD62L^+^CD44^-^, CD62L^+^CD44^+^ and CD62L^-^CD44^+^ CD8 T cells in the iLN following primary and secondary MC38 tumor challenges**. Naive-like (CD62L^+^CD44^-^), central memory-like (CD62L^+^CD44^+^), and effector-like (CD62L^-^CD44^+^) CD8 T cells were examined in iLNs of RARα-KO mice following primary and secondary MC38 tumor challenges. Representative and combined data are shown. Data are means ± SEM. n ≥ 4 mice. Statistical significance was determined using one-way ANOVA with Tukey’s multiple comparison test. *P ≤ 0.05; **P ≤ 0.01; ***P ≤ 0.001; ****P ≤ 0.0001.

**Figure S8. tSNE immune cell clusters in the scRNA-seq data**. (A) Expression of *Cd3e, Cd8a, Cd4* and *Foxp3* in the tSNE plot. (B) Marker genes that identify cell clusters in the scRNA seq data. Cells in C1 express genes encoding markers for regulatory T cells (*Foxp3,* Il2ra). Cells in C2 expressed genes encoding markers of naïve and central/early memory T cells (*Tcf7, Sell, S1pr1,* and *Ccr7*). C3 cells had decreased expression of the naïve T cell-associated genes but increased expression of effector-associated genes (*Cd44, Gzmb, Gzmk, Prf1,* and *Irf8*). C3 cells also had expression of genes associated with T cell exhaustion (*Pdcd1, Tigit, Lag3,* and *Havcr2*). Effector and exhaustion markers were further increased in C4 and C5. C6 cells expressed genes typically expressed by effector memory CTLs (*Xcl1, Ifng, Ccl3, Ccl4, Irf4, Nr4a1,* and *Ccr7).* C7 cells expressed cell cycle genes (*Mki67, Stmn1, Top2a, Birc5*). Markers for myeloid cells (*Lyz2, Itgam, Fcgr1,* and *Cd14)* were expressed by C8 and epithelial markers (*Col3a1, Dcn, Gsn*) were expressed by C9 and C10, while C10 cells also showed the expression of mitotic genes as in C7. C11 cells highly express *mt-Atp8* and *mt-Cytb*, which are associated with apoptosis. (C) Expression of *Rara, Rarb and Rarg* in the tSNE plot retrieved from the scRNA-seq data.

**Figure S9. Expression of effector molecules by CTLs in tumors.**

(A) Expression of *Ifng, Gzmb* and *Prf1* by CTLs in MC38 tumors of WT, RARα-KO, and RARα-TG mice. The data were from scRNA-seq analysis of T cells in MC38 tumors.

(B) Expression of IFNγ, CCL5, GzmB, and perforin by tumor-infiltrating CTLs. Data are means ± SEM (n ≥ 5 mice). Statistical significance was determined using one-way ANOVA with Tukey’s multiple comparison test (B).

**Figure S10. A CellChat systematic bioinformatics analysis for potential cellular interactions in MC38 tumors of WT, RARα-KO, or RARα-TG mice.** (A) A set of CellChat diagrams identifying major outgoing and incoming signaling networks among the cell clusters in MC38 tumors of WT, RARα-KO and RARα-TG mice. (B) Violin plot showing the expression of *Ccl3, Ccl4*, and *Ccl5* by CTLs from the MC38 tumors of WT, RARα-KO, or RARα-TG mice. (C) Violin plots showing the relative expression of *Ccr1* and *Ccr5* in myeloid subclusters. (D) Violin plot showing the relative expression of *Ccr5* in distinct clusters of CTLs from WT, RARα-KO and RARα-TG. (E) Chord diagrams showing significant interactions among the clusters for the *Ccl3-Ccr5, Ccl4-Ccr5*, and *Ccl5-Ccr5* chemokine-receptor pairs. The thickness of the lines corresponds to the strength of the predicted interaction. For the analysis, the scRNA seq data obtained from MC38 tumor-infiltrating T cells in WT, RARα-KO, and RARα-TG mice (Fig. 4) was used.

**Figure S11. Expression of major trafficking receptors and effector molecules in CTLs.** Expression of major trafficking receptors by Teff CTL clusters in MC38 tumors of WT, RARα-KO and RARα-TG mice. The heatmap shows mRNA expression levels for major trafficking receptors. The data was retrieved from the scRNA-seq data described in Figure 4.

**Figure S12. Impact of T cell-expressed RARα on the expression of transcription factors in CTLs.** (A) Pathways predicted to be increased in RARα-KO and RARα-TG CD8 T cells based on up-regulated genes. Significantly up-regulated (≥1.5 folds) genes from the scRNA-seq data were processed by the DAVID Functional Annotation analysis. Expression of IRF4 (B) and TCF1 (C) by tumor-infiltrating CTLs in MC38 and B16 tumors of WT, RARα-KO, and RARα-TG mice. Data are means ± SEM. n =6-12 mice. Statistical significance was determined using one-way ANOVA with Tukey’s multiple comparison test (B, C). **P* ≤ 0.05; ***P* ≤ 0.01; ****P* ≤ 0.001; *****P* ≤ 0.0001.

**Figure S13. T cell-expressed RARα affects the myeloid cells in tumors**. (A) Myeloid sub-cell clusters within the tSNE C8 cluster. (B) Major marker genes of myeloid clusters. (C) tSNE plots for myeloid cells in MC38 tumors of WT, RARα-KO, and/or RARα-TG mice. (D) Frequencies of predicted myeloid cells among all captured cells in MC38 tumors of WT, RARα-KO, and/or RARα-TG mice. (E) Predicted ratios of M1 to M2 macrophages in MC38 tumors. (F) DEGs of monocytes and macrophages in MC38 tumors of WT, RARα-KO, and RARα-TG mice. (G) Violin plots for selected genes expressed in tumor-infiltrating macrophages from the three groups of mice. (H, I) Pathway analysis for the myeloid DEGs. Pathways predicted to be increased in RARα-KO and RARα-TG CTLs based on up-regulated genes are shown. Significantly up-regulated (≥1.5 folds) genes from the scRNA-seq data were processed by the DAVID Functional Annotation analysis.

**Figure S14. RARα-deficiency improves *in vitro* CAR T cell cytotoxicity**. (A) Exemplary images for the co-culture of CAR CTLs with MC38-hCD19 tumor cells at 0.5 ratio. Caspase-positive MC38 cells (size ≥ 150 µm^2^) are shown in red. (B) Representative of cytotoxic activity of WT, RARα-KO, and RARα-TG CAR T cells against MC38-hCD19 cells. CAR-T cells and tumor cells were co-cultured at 1:1 ratio. Caspase-positive tumor cells (size ≥ 150 µm^2^) are shown in purple. (C) Quantification of MC38-hCD19 killing over time at different ratios. n≥5 wells over two independent experiments. Error bars denote SEM.

**Figure S15**. **Selected genes that have peaks for the binding of RARα and BATF on the CTL chromatin in RA-depleted (control) and replete (RA) conditions.** (A) Comparison between RA inducibility (X axis) and RARα binding (Y axis) activities. Fold induction of gene expression by RA determined by RNA-seq and RARα binding activity determined by ChIP-seq in CTLs were plotted. (B) Selected genes that have both RARα binding activity and RA inducibility. RARα and BATF peaks are shown on these genes in CTLs cultured in the RA deplete and replete conditions. (C) Three groups of genes in terms of RARα and BATF binding activity. ChIP-seq data are shown for CTLs that are cultured in RA-depleted and RA (15 nM)-containing conditions. Integrated genome browser (IGB) was used to display the binding activity of RARα and BATF on the chromatin regions of interest.

**Figure S16. Inhibition of the p300 HAT decreases the trafficking receptor switch in CTLs.** OT-1 T cells were activated for 2 days with the SIINFEKL peptide, IL-7 and IL-15 in the presence or absence of the HAT inhibitors (C646 and anacardic acid/AA at 5 μM). The expression of CCR7 and CXCR3 and the frequencies of CCR7^+^ CXCR3^-^, CCR7^+^ CXCR3^+^, and CCR7^-^ CXCR3^+^ CTLs were determined by flow cytometry. Data are means ± SEM. Statistical significance between two groups was determined by Student’s paired T-test (B). **P* ≤ 0.05; ***P* ≤ 0.01; ****P* ≤ 0.001; *****P* ≤ 0.0001.

**Figure S17**. **A proposed model for the negative role of RARα in regulating anti-tumor Teff CTLs.** RARα is highly expressed by differentiated effector and exhausted T cells. RARα exerts a negative effect on Teff differentiation and the associated trafficking receptor switch. Upregulated RARα levels decrease the nuclear HAT activity in CTLs, and this suppresses the expression of key Teff transcription factor BATF and trafficking receptors potentially important for migration into tumors.
